# Upcycling agro-waste into sustainable bioenergy: using carbonized cabbage core wastes bio-anodes and novel bacterial insights in MFCs for sugarcane wastewater treatment

**DOI:** 10.1186/s12934-026-02954-7

**Published:** 2026-03-02

**Authors:** Ahmed Abotaleb, Mai Ramadan, Dina H. Amin, Alaa F. Elsayed, Nasser A. M. Barakat, Amina Shaltout, Abeer El Shahawy

**Affiliations:** 1 Department of Civil Engineering, Faculty of Engineering, Suez Canal University, P.O. Box 41522, Ismailia, Egypt; 2 Department of Microbiology, Faculty of Science, Ain Shams University, Cairo, 1566 Egypt; 3 Chemical Engineering Department, Faculty of Engineering, Minia University, Minya, 61519 Egypt; 4 Chemical Engineering Department, Faculty of Engineering, Atatürk University, 25240 Erzurum, Turkey

**Keywords:** Bio-anodes, Sustainable energy, Microbial fuel cells (MFCs), Biomass, Electroactive bacteria, Exiguobacterium, Extracellular electron transfer, Sugarcane wastewater

## Abstract

In pursuit of sustainable energy and waste valorization, this study introduces a novel bio-anode synthesized from carbonized cabbage core waste, a low-cost, abundant agricultural byproduct engineered for high-performance microbial fuel cells (MFCs) utilizing sugarcane wastewater as a bio-electrochemical substrate. Carbonization at moderate temperatures yielded a porous, honeycomb-like carbon architecture conducive to microbial attachment, as confirmed via scanning electron microscopy (SEM). X-ray diffraction (XRD) revealed partial graphitization within a disordered matrix, optimizing conductivity while maintaining structural porosity. When applied in a dual-chamber MFC, the cabbage-derived bio-anode achieved a remarkable power density of 904.1 mW/m², exceeding that of conventional carbon felt by over 21-fold, alongside a maximum open-circuit voltage (OCV) of 822.4 ± 5.2 mV (2.33× increase). Critically, microbial analysis of the biofilm revealed a dominant presence of *Exiguobacterium*, marking the uniqueness of *Exiguobacterium aurantiacum* associated with anode biofilm during sugarcane-wastewater-driven MFC operation. Its tolerance to high-sugar environments and its putative role in extracellular electron transfer (EET) expand the known spectrum of bioelectrogenic species and introduce new possibilities for strain engineering in bioenergy research. This integrated approach exemplifies circular economy principles, converting agricultural and agro-industrial waste into clean electricity while advancing decentralized wastewater treatment. The outcomes of this work support multiple UN Sustainable Development Goals: including SDG 6 (Clean Water and Sanitation), SDG 7 (Affordable and Clean Energy), SDG 12 (Responsible Consumption and Production), and SDG 13 (Climate Action). By coupling waste-derived electrode materials with novel microbial pathways, this research advances the development of low-cost, scalable bioenergy systems for sustainable rural and industrial applications.

**Supplementary Information:**

The online version contains supplementary material available at 10.1186/s12934-026-02954-7.

## Introduction

Water pollution and the energy crisis are currently among the most prevailing problems facing human beings and are promoting other issues like climate change. This has attracted the world’s attention to the importance of searching for sustainable, eco-friendly, and environmentally friendly solutions for those problems [1]. To tackle these problems, researchers have placed an emphasis on renewable energy sources, such as wind and solar energy. Wastewater is considered one of the most abundant sources of renewable energy, as it contains approximately 4–10 times the energy required to treat it [2, 3]. Microbial electrochemical cells (MECs) are a promising sustainable technology that uses active extracellular electrogenic microorganisms as catalysts to convert the chemical energy stored in organic pollutants in wastewaters into electric energy (in a microbial fuel cell setup) or biogas (in a microbial electrolysis cell) [4]. Unlike other conventional electrochemical devices, MESs can operate using a very wide range of fuels and simultaneously treat wastewater [5]. However, the limited ability of microorganisms to attach to the anode surface results in low power generation, which in turn prevents their use in practical applications [6]. The anode is a crucial part of a microbial fuel cell, as it significantly affects the ability of microorganisms to adhere to the electrode surface and the efficiency of extracellular electron transfer [7]. A good anode should have high biocompatibility, high conductivity, high active electrochemical area, good mechanical properties, and chemical stability [8]. Carbon materials are currently the most widely employed materials as anodes in microbial fuel cells owing to their biocompatibility, high surface area, good electrical and mechanical properties, and low cost [9]. Therefore, numerous researchers have studied the design of new carbon-based anodes or the modification of existing ones to enhance the performance of microbial fuel cells and increase overall energy harvesting. Moreover, metal-based electrodes such as nickel, iron, copper, stainless steel, and titanium have been investigated as potential anodes in microbial fuel cells; however, they possess high biotoxicity and a high corrosion possibility, which makes them unfavorable anodes for microbial fuel cells [10].

Additionally, carbon-based anodes can be further categorized into two-dimensional (2D) anodes, such as carbon cloth and carbon paper, and three-dimensional (3D) anodes, such as carbon brushes, carbon foam, and graphite rods [11]. It’s noteworthy that 3D anodes outperform 2D anodes in microbial fuel cells due to the presence of macro- and micro-pores in their structure, which allows more microorganisms to attach to the anode surface and facilitates substrate and ion diffusion into the electrode’s inner pores [12]. Consequently, researchers have focused on studying 3D carbon-based anodes, such as hydrogels, carbon foam, and carbon nanoporous materials, to replace commercial 2D anodes [13]. One of the most used techniques is direct carbonization of carbon-based waste, especially agricultural waste [14]. Yuan et al. used carbonized loofah sponge at 900 °C and achieved a maximum power density of 1090 ± 72 mW m^−2^ [15]. Chen et al. carbonized chestnut shell at 900 °C and used it as an anode in a single chamber MFC, the cell demonstrated a maximum power density of 759 ± 38 mW m^−2^ [16]. Chen et al. used activated carbonized chestnut shells as an anode in an air-cathode MFC and achieved a maximum power density of 850 mW m^−2^ [17]. Yang et al. carbonized cocklebur fruit at 900 °C and used it as an anode in a single chamber MFC; the maximum power density was 572 ± 24 µW m^−3^ [18]. Ma et al. carbonized municipal sludge at 800 °C and used it as an anode in a double chamber MFC; the cell achieved a maximum power density of 568.5 mW m^−2^ [19]. Thipraksa et al. pyrolyzed coconut shells at 400 °C and used them in a double chamber MFC; the cell demonstrated a maximum power density of 283.42 ± 5 mW m^−2^ [20]. The advantage of using agricultural waste as a carbon precursor for direct carbonization is that it contains plant heteroatoms, such as nitrogen, sulfur, phosphorus, silicon, and trace heavy metals, which act as natural dopants. This helps in producing self-doped materials that can enhance the performance of microbial fuel cells [21–23]. Moreover, the graphitization of natural polymers yields very high surface area carbonaceous electrodes due to the formation of very high amounts of macro and micropores [24].

In this study, a simple direct carbonization process is employed to convert cabbage core waste, an abundant and underused agricultural waste, into a high-performance anode for a two-chamber microbial fuel cell, which is used to treat industrial wastewater. The electrical performance and COD removal were recorded, and the anode morphology, chemical structure, and bacterial consortia were studied for the assembled anodes. Moreover, two different types of wastewater were investigated to demonstrate the effect of using wastewater as an anolyte.

## Materials and methods

### Materials

Plain carbon felt (CC) was purchased from Fuel Cell Store in the USA. A phosphate buffer solution was purchased from Power Chemical. Egypt. Potassium ferricyanide was bought from ADVENT Chembio. Cabbage stalk waste was obtained from a local market in Egypt and used without further treatment.

### Wastewater characteristics

The anolyte was sourced from the wastewater of a nearby sugarcane milling business. As stated in Table 1, the water properties were measured using methods established by the American Public Health Association (APHA) for the evaluation of water and wastewater.

**Table 1:** Water quality criteria and their measurement methods

Wastewater quality parameter	Standard method	Value	References
pH	Benchtop pH meter (Adwa AD 1030)	5.5	[25]
BOD	Incubation at 20 °C for 5 days	22,500	
Total suspended solid	Dried at 103–105 °C	56,840	
COD	The V-730 Double Beam UV-Visible Spectrophotometer is used in the Closed Reflux technique	72,000	
Total dissolved solid	Dissolved solids evaporated at 103–105 °C	33,440	
Color	Portable pH/ORP Meter (HI8424) Digital Colorimeter	13,600	

### Anode preparation

**Fig. 1:**
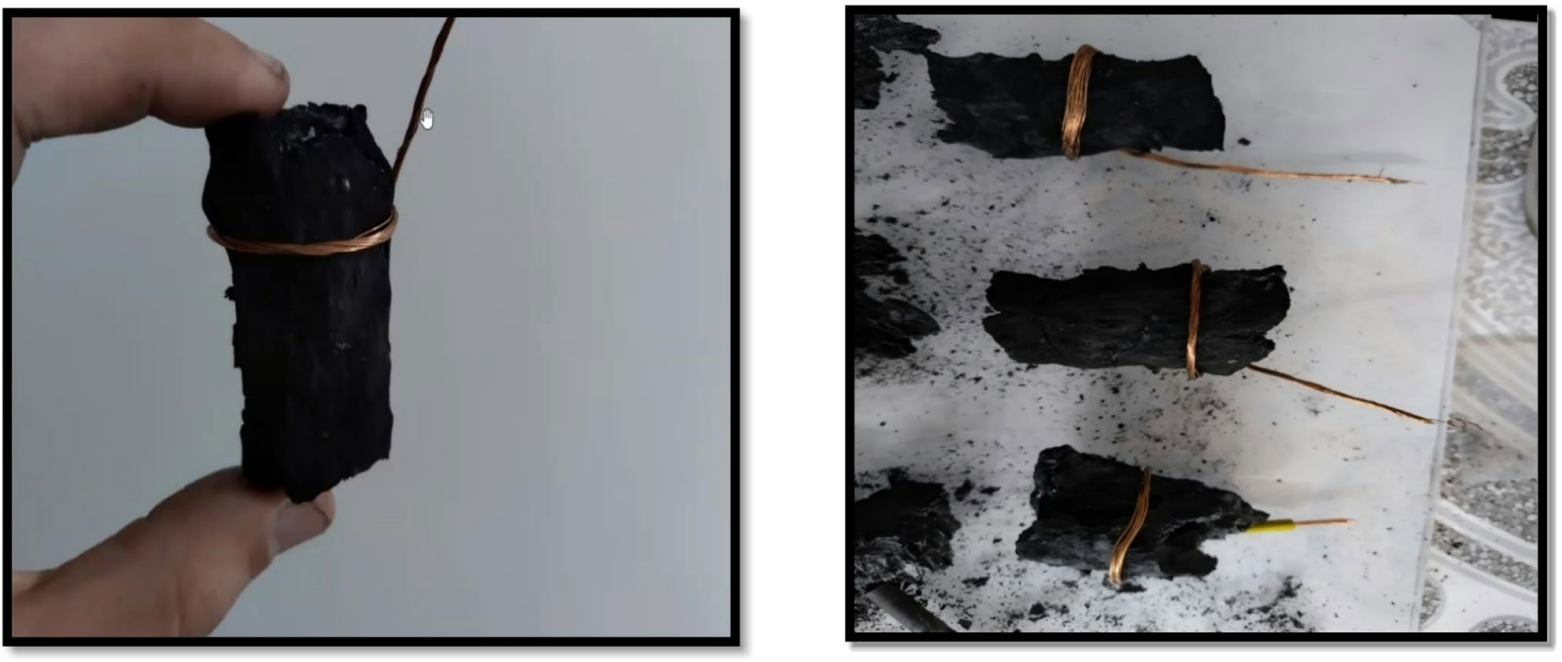
The cabbage core sample after carbonization at 800 °C

Three pieces of cabbage core waste were collected from a local market, washed with water to remove impurities, dried at 80 °C, and placed in a tube furnace. The chamber was sealed tightly and vacuumed to create an oxygen-free environment. The carbonization process was carried out at a heating rate of 2 °C/min and held for 2 h at a temperature of 800 °C in a vacuum pressure of − 100 kPa. The weights of the 3 replicates were 86.9, 80.5, and 85 g before carbonization. After carbonization, the samples were left to cool down naturally inside the furnace. The carbon yield of the samples was 24 ± 1.5%, as shown in Fig. 1. The prepared carbon anode was wrapped with a copper wire to serve as a current collector.

### MFC construction and operation

**Fig. 2:**
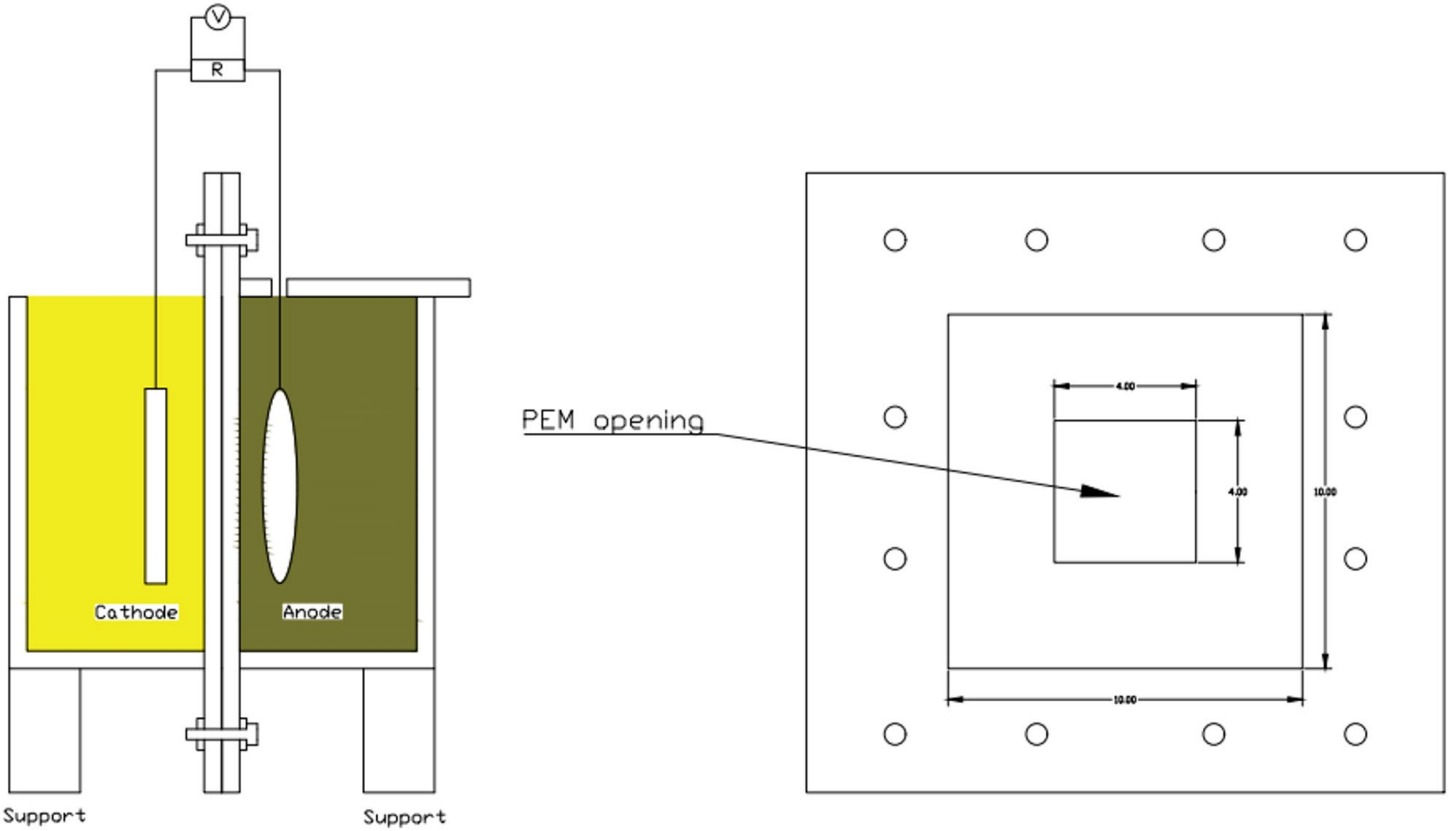
Double-chamber microbial fuel cell design

The study was conducted in batch mode using a double-chamber microbial fuel cell setup, as illustrated in Fig. 1. The cell body was constructed by connecting the anode and cathode chambers (500 mL chambers with dimensions of 5 cm × 10 cm × 10 cm) via a proton exchange membrane. Sulfonated polystyrene with a thickness of 250 μm was prepared as described in previous literature [26] and used as the proton exchange membrane. The as-prepared anode was immersed in the anolyte in the anode chamber. For comparison, a piece of carbon felt with dimensions of 4 cm × 4 cm was used as the commercially available anode. A piece of carbon felt with dimensions of 4 cm × 4 cm was used as a cathode in all assembled microbial fuel cells. The cathode chamber was filled with potassium ferricyanide (K_3_[Fe(Cn)_6_] (50mM) in PBS solution (50 mM). Microbial fuel cells were operated at room temperature (27 °C) for 7 days, during which the anode chamber was kept tightly closed to maintain an anaerobic condition. An Arduino-based data logger was used to record the open-circuit voltage at one-hour intervals. Connecting the anode’s current collector and cathode with a Potentiostat/Galvanostat (AMEL Model 2550) was used to close the cell circuit once the open-circuit voltage had stabilized. Connecting the working electrode to the anode current collector and both the counter and reference electrodes to the cathode current collector established the connection. With the greatest stable open-circuit voltage (OCV) measurement acting as the starting potential value and zero as the final potential value, linear sweep voltammetry (LSV) was performed. All assembled cells had their (I–V) curves recorded after LSV, which was conducted at a scan rate of 1 mV/s. A scan rate of 1 mV/s was used for the cyclic voltammetry, which covered a voltage window of − 1000 mV to 200 mV. The outer surface area of the proposed anodes was calculated based on assuming a cylindrical surface with a diameter equal to the average diameter of the sample. Current densities are normalized to the outer surface area of each anode (Fig. 2).

### Characterization

The morphology of the prepared anodes’ surfaces and the identification and quantification of surface elements were investigated using a scanning electron microscope (SEM) equipped with an energy-dispersive spectrometer (JSM-IT210). The structure and chemical composition of the prepared samples were investigated using an X-ray diffraction technique (XRD) on a Bruker D2 Phaser Advance diffractometer equipped with Cu Kα radiation of wavelength λ = 0.15406 nm in a 2θ range from 10° to 80° with a step size of 0.02 ° and a time per step of 0.2 s. A Shimadzu 8300 spectrometer was used to perform Fourier transfer analysis within a spectral range of 400–4000 cm^−1^. With 100 mg KBr, the samples were ground into a fine powder. It was then compacted for 5 min under seven tons into a thin pellet.

Surface area, pore size, and pore volume were measured by the Brunauer–Emmett–Teller (BET) analyzer model Nova Touch LX2, manufactured by Quanta Chrome Company in the USA. The measurements are done using the standard volumetric method by nitrogen adsorption at 77 °K.

### Bacterial isolation and analysis

Bacterial isolates were obtained from the anode biofilm of the microbial fuel cell (MFC) after seven days of stable operation with sugarcane wastewater (see “MFC construction and operation” section for MFC setup). Biofilm samples were aseptically scraped from the carbonized cabbage-core anode surface and streaked on nutrient agar to recover distinct colonies. Four similar representative isolates were purified through repeated sub-culturing to ensure single-colony purity and coded as S1–S4 based on the chronological order of isolation. After identification, pure cultures were preserved by mixing with sterile glycerol (30% v/v) and stored in Eppendorf tubes at −20 °C for long-term maintenance.

#### Biochemical identification using VITEK

Microorganism identification of the selected strains (S4–S1) was performed using the VITEK^®^ MS system (bioMérieux, France) based on Matrix-Assisted Laser Desorption Ionization Time-of-Flight Mass Spectrometry (MALDI-TOF MS) (bioMérieux, 2025). Isolates were sub-cultured on appropriate agar media and incubated under optimal conditions. Freshly grown colonies were applied onto a VITEK^®^ MS-DS target slide. All technical details are provided in Supplementary Material S1.

#### Molecular identification of the selected bacterial strains

Genomic DNA was extracted from bacterial samples (S1–S4) using the QIAamp DNA Mini Kit (QIAGEN, Germany), following the manufacturer’s protocol. Polymerase Chain Reaction (PCR) amplifications were performed using the Emerald Amp GT PCR Master Mix (2× premix) (Takara, Code No. RR310A) according to the manufacturer’s protocol, using the primers listed in Table 2.

**Table 2:** The primers used in our study

Gene	Primer	Primer sequence 5ʹ-3ʹ	Amplified product	References
16S rRNA	F27	AGAGTTTGATCMTGGCTCAG	1485 bp	[27]
	R1492	TACGGYTACCTTGTTACGACTT		

Agarose gel electrophoresis was performed as described by [28] with minor modifications. All technical details are provided in Supplementary Material S1.

The 16 S rRNA gene partial sequences obtained in this study were deposited in the NCBI GenBank database. The accession numbers are as follows: *Klebsiella variicola* strain S1(GenBank: PX218691) and *Exiguobacterium aurantiacum* strain S4 (GenBank: PX218690). https://www.ncbi.nlm.nih.gov/nuccore/3048505901, and, https://www.ncbi.nlm.nih.gov/nuccore/3048505900.

#### Phylogenetic analysis of the selected bacterial strains

The phylogenetic tree of S1 and S4 strains was constructed based on 16 S rRNA gene sequences to determine their evolutionary relationships. The sequences were obtained from publicly available databases and aligned using the MUSCLE algorithm [29] in Geneious Prime software (https://www.geneious.com/series/multiple-alignments). The aligned sequences were then used to generate a phylogenetic tree using the FastTree option in Geneious software. FastTree computes approximately maximum-likelihood phylogenies with efficient heuristics [30], providing reliable branch support values. The tree topology was assessed, and branch support values (posterior probabilities) were indicated at the nodes, with values closer to 1 signifying stronger statistical confidence in the branching patterns. To enhance the visual interpretation of the phylogeny, different clades were color-coded to distinguish major taxonomic groupings. The scale bar represents genetic distance (0.005), illustrating the degree of sequence divergence among the strains. The final tree was exported and annotated for presentation.

## Results and discussion

### Characterization of obtained anodes

#### X-ray diffraction (XRD)

**Fig. 3:**
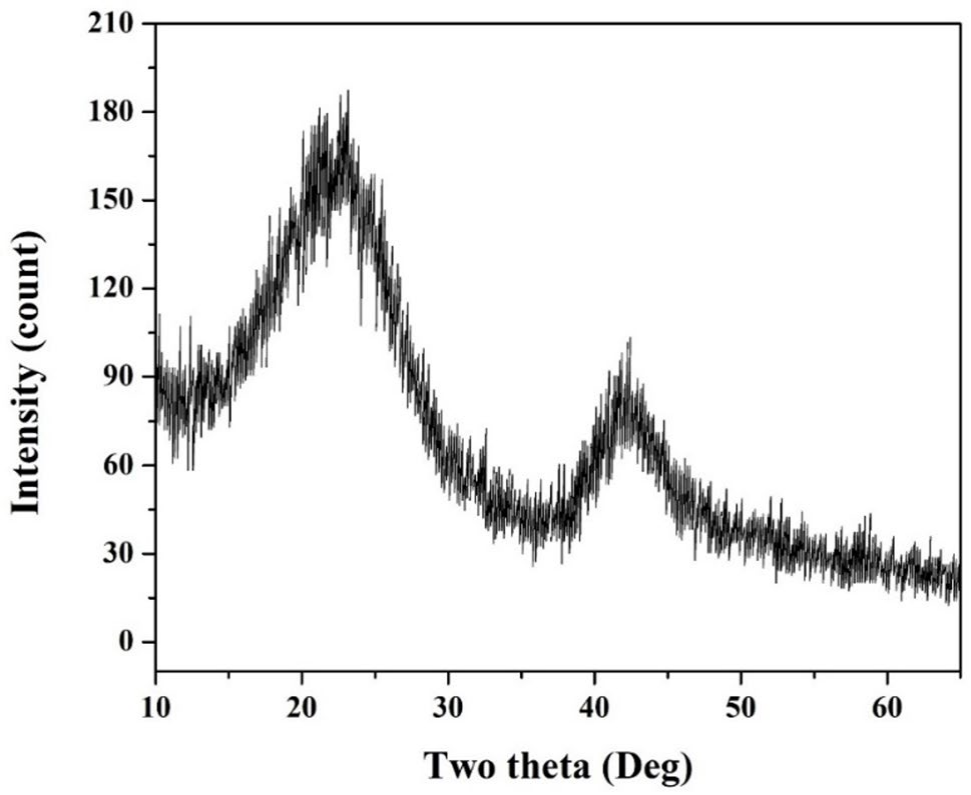
XRD pattern for the graphitized cabbage stalk

The graphitized cabbage stalk anode was characterized using X-ray diffraction (XRD) to investigate its structural properties and confirm the successful carbonization and graphitization of the material (Fig. 3). The XRD pattern revealed two broad peaks at 2θ values of approximately 22.5° (high intensity) and 42° (lower intensity). The first peak is indicative of the (002) plane of graphitic carbon, which corresponds to the stacking of graphene layers in a disordered or turbostratic structure. The broad nature of the peak suggests the presence of amorphous carbon or partially graphitized carbon, which is typical for biomass-derived carbon materials. The high intensity of this peak indicates a significant degree of carbonization, where the organic components of the cabbage stalk have been converted into carbonaceous material. The broadness of the peak also implies a relatively low degree of crystallinity and the presence of defects or disordered carbon structures. This is consistent with the carbonization process at 800 °C, which is sufficient to decompose organic matter but may not fully achieve the highly ordered crystalline structure of pure graphite. The disordered carbon structure can be advantageous for microbial fuel cell (MFC) applications, as it provides a high surface area and active sites for microbial colonization and electron transfer.

The second peak at 2θ ≈ 42° corresponds to the (100) plane of graphitic carbon, which is associated with the in-plane structure of graphene layers. The lower intensity of this peak compared to the (002) peak suggests that the graphitization process is incomplete, and the material retains some degree of disorder. The presence of this peak, however, confirms the formation of graphitic domains within the carbonized material. The (100) peak is characteristic of the hexagonal arrangement of carbon atoms within the graphene layers. Its lower intensity indicates that the graphitic domains are smaller or less ordered compared to highly crystalline graphite. This partial graphitization is expected for biomass-derived carbon materials, as the carbonization temperature of 800 °C is below the threshold required for complete graphitization (typically > 2500 °C).

The XRD results demonstrate that the graphitized cabbage stalk possesses a mixed structure of amorphous and graphitic carbon, which is highly desirable for anode materials in microbial fuel cells. The disordered carbon regions provide a high surface area and porosity, facilitating microbial adhesion and biofilm formation. Meanwhile, the graphitic domains enhance electrical conductivity, enabling efficient electron transfer from electroactive bacteria to the anode surface.

The presence of defects and functional groups on the carbon surface, as indicated by the broad XRD peaks, can further enhance the anode’s performance by providing additional active sites for redox reactions and improving the interaction between the anode and the microbial community. The combination of these structural features makes the graphitized cabbage stalk a promising and sustainable anode material for microbial fuel cell (MFC) applications.

#### Fourier-transform infrared spectrum (FTIR)

**Fig. 4:**
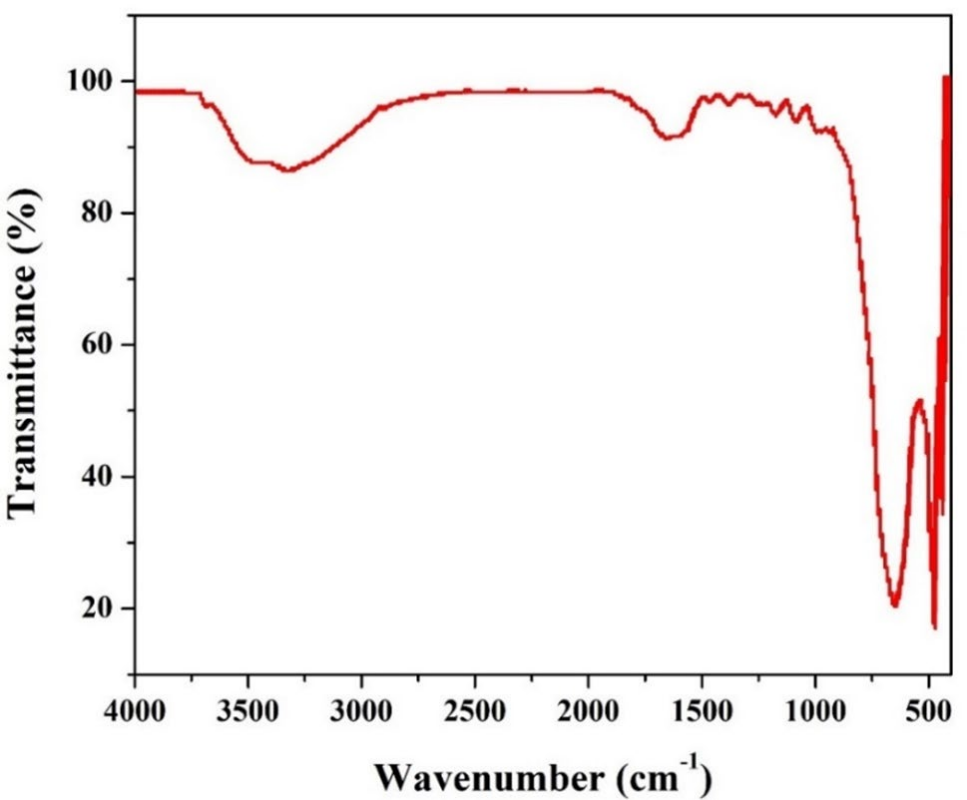
FTIR spectrum for the graphitized cabbage stalk

The Fourier-transform infrared (FTIR) spectrum of the graphitized cabbage stalk anode provides valuable information about the functional groups and chemical bonds present on the material’s surface. These functional groups play a critical role in determining the electrochemical properties and microbial interactions of the anode in microbial fuel cells. The broad peaks at 3450 cm^−1^ and 3310 cm^−1^ are attributed to O–H stretching vibrations, which are typically associated with hydroxyl groups (OH) present on the surface of the material [31]. The broad nature of these peaks (Fig. 4) suggests the presence of adsorbed water molecules or hydrogen-bonded hydroxyl groups, which are common in carbonized biomass materials. The presence of hydroxyl groups is beneficial for MFC applications, as they enhance the hydrophilicity of the anode surface, promoting microbial adhesion and biofilm formation [32]. Additionally, these groups can participate in redox reactions, facilitating electron transfer between the microbes and the anode [33]. The relatively broad peak at 1630 cm^−1^ corresponds to C=C stretching vibrations in aromatic rings or conjugated systems, which are characteristic of graphitic carbon structures. It may also include contributions from C=O stretching vibrations in carbonyl groups (e.g., quinones, ketones, or carboxylic acids) that are often present on the surface of carbonized materials. The presence of C=C bonds confirms the formation of graphitic domains during the carbonization process, consistent with the XRD results [34]. The C=O groups, if present, can act as active sites for electrochemical reactions, further enhancing the anode’s performance in MFCs.

The sharp decrease in transmittance from 905 to 660 cm^−1^, with a minimum of 660 cm^−1^, is indicative of C–H out-of-plane bending vibrations in aromatic hydrocarbons [35]. This region is characteristic of substituted benzene rings or polycyclic aromatic hydrocarbons (PAHs), which are common in carbonized biomass. The low transmittance (20%) at 660 cm^−1^ suggests a high concentration of aromatic structures, which are typical of carbon materials derived from biomass pyrolysis. These aromatic structures contribute to the electrical conductivity of the material, making it suitable for use as an anode in microbial fuel cells (MFCs) [36]. The sharp peak at 470 cm^−1^ is likely associated with C–C bending vibrations or metal-oxygen (M–O) vibrations if any metal impurities are present in the sample. In the context of carbonized biomass, this peak is often attributed to the presence of inorganic residues or ash content that may have remained after the carbonization process [37]. The low transmittance at this wavelength indicates strong absorption, which could be due to the presence of inorganic compounds or highly ordered carbon structures. While inorganic residues are generally undesirable, their presence in small amounts may not significantly affect the anode’s performance in MFCs.

The FTIR spectrum of the graphitized cabbage stalk anode reveals a complex surface chemistry with a combination of functional groups that are highly advantageous for MFC applications. The presence of hydroxyl groups enhances the hydrophilicity of the anode surface, promoting the attachment of electroactive bacteria and the formation of a stable biofilm. This is critical for efficient electron transfer in MFCs. The C=O and C=C functional groups provide active sites for redox reactions, facilitating electron transfer between the microbial community and the anode. These groups can also participate in pseudocapacitive charge storage, improving the overall performance of the MFC. The aromatic and graphitic structures (660 cm⁻¹ and 1630 cm⁻¹) contribute to the electrical conductivity of the anode, ensuring efficient electron transport from the bacteria to the external circuit. While the presence of inorganic residues (470 cm⁻¹) is not ideal, their impact on the anode’s performance is likely minimal if present in small amounts. The surface defects associated with these residues may even provide additional active sites for microbial interactions.

#### Scanning electron microscope (SEM)

**Fig. 5:**
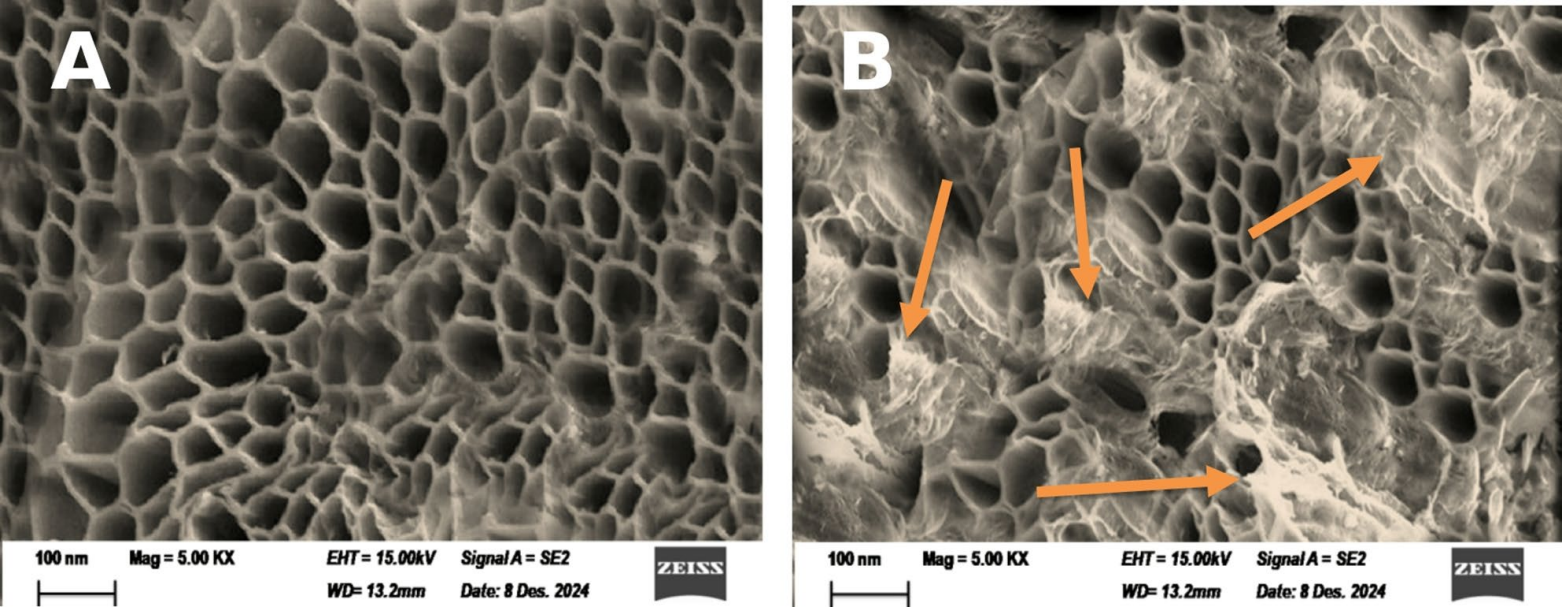
SEM image for the graphitized cabbage stalk before (**A**) and after utilization as anode in the MFC (**B**)

The scanning electron microscopy (SEM) images of the fresh (Fig. 5A) and used (Fig. 5B) cabbage stalk anode provide critical insights into the morphological characteristics of the material and its interaction with electroactive microorganisms in the microbial fuel cell. The comparison between the fresh and used anode reveals significant changes in surface morphology, confirming the successful colonization of microorganisms and highlighting the suitability of the graphitized cabbage stalk as an anode material for microbial fuel cells (MFCs).

The SEM image of the fresh anode reveals a highly porous, honeycomb-like structure, which is characteristic of carbonized biomass materials. The pores are uniformly distributed across the surface, with sizes adequate for the accommodation of microorganisms. This porous structure is a direct result of the carbonization process, which removes volatile organic compounds and leaves behind a carbon-rich, interconnected network of pores. The high porosity and large surface area of the fresh anode are advantageous for MFC applications, as they provide ample space for microbial colonization and biofilm formation. The pores also facilitate the diffusion of nutrients and metabolites, ensuring a favorable environment for the growth and activity of electroactive bacteria.

The honeycomb structure is particularly beneficial because it allows microorganisms to penetrate deep into the anode, increasing the effective surface area for electron transfer. This three-dimensional architecture enhances the anode’s performance by maximizing the contact between the microbes and the conductive carbon material.

The SEM image of the used anode clearly shows the attachment of microorganisms on the surface of the electrode. The microbial cells appear as small, spherical, or rod-shaped structures covering the anode surface, confirming the successful colonization of electroactive bacteria. In addition to the visible microbial cells on the surface, it is expected that a numerous population of microorganisms resides inside the deep pores of the anode. This is supported by the honeycomb-like structure observed in the fresh anode, which provides an ideal habitat for microbial growth. The microorganisms inside the pores are likely to play a significant role in the electrochemical performance of the MFC, as they contribute to the overall electron transfer process.

The biofilm formation on the anode surface is a critical factor in MFC performance, as it facilitates direct electron transfer from the microorganisms to the anode. The presence of a dense microbial community also indicates that the anode material is biocompatible and provides a suitable environment for the growth and activity of electroactive bacteria.

The honeycomb-like structure of the fresh anode provides a high surface area and ample space for microbial colonization. This enhances the anode’s capacity to host a large population of electroactive bacteria, which is essential for efficient electron transfer and high power output in MFCs. The attachment of microorganisms on the surface and within the pores of the used anode confirms the material’s ability to support biofilm formation. The biofilm acts as a conductive bridge between the bacteria and the anode, facilitating direct electron transfer and improving the overall performance of the MFC. The interconnected, porous structure enables the efficient diffusion of nutrients and metabolites, creating a favorable environment for microbial growth and activity. This is particularly important for the long-term operation of MFCs, as it prevents the accumulation of waste products that could inhibit microbial activity.

The successful colonization of microorganisms on the anode surface demonstrates the biocompatibility of the graphitized cabbage stalk material. Additionally, the use of agricultural waste (cabbage stalk) as a precursor for the anode aligns with the principles of sustainability and circular economy, making it an environmentally friendly alternative to conventional anode materials.

#### BET results

**Fig. 6:**
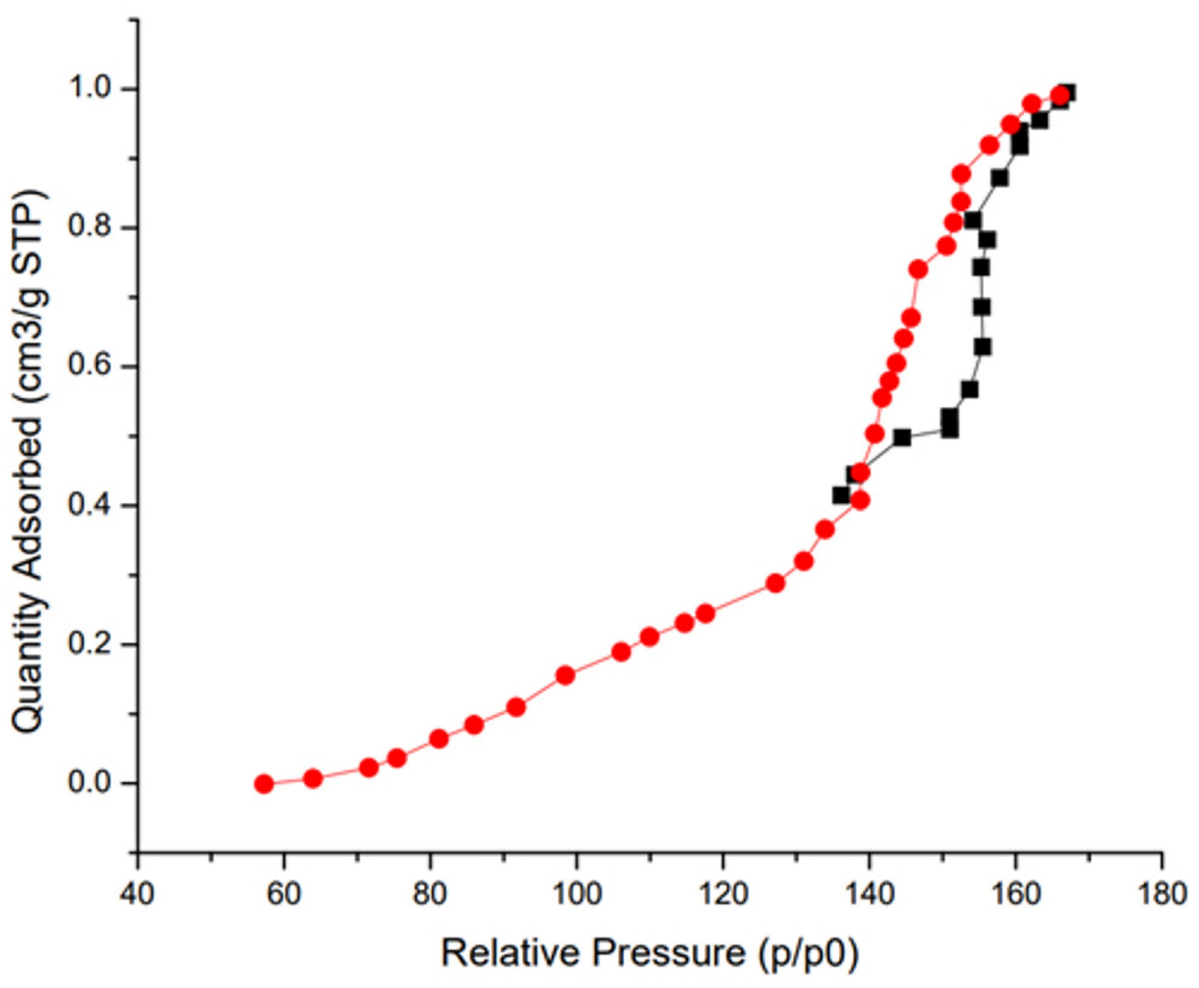
Adsorption–desorption curve of the carbonized cabbage stalk

The adsorption-desorption curve for the carbonized cabbage stalk sample exhibits a Type IV isotherm with an H3 hysteresis loop (typical for mesoporous materials). The curve’s adsorption branch indicates moderate uptake at low relative pressures (P/P0 < 0.1), associated with micropore filling, followed by a sharp increase at higher relative pressures due to multilayer adsorption and capillary condensation in mesopores. Samples exhibited BET surface area of 178.5 m^2^/g, a pore volume value of 0.21 cm^3^/g, and an average pore diameter of 3.5 nm. The small surface area can be attributed to the low carbonization temperature; however, it’s noteworthy that raising the carbonization temperature to 900 °C led to the formation of significant cracks in several locations in the sample, which made its structure unstable and fragile (Fig. 6).

### Performance of the assembled MFCs and electrochemical analysis

The experiment aims to make the cell as cost-effective as possible. Therefore, the carbonization temperature was maintained at a moderate level (800 °C), and the proton exchange membrane was fabricated from waste Styrofoam using a simple sulfonation process. To evaluate the performance of the prepared anode, its performance was compared to that of carbon felt, one of the most well-known commercially available carbon materials in fuel cells. Carbon felt is considered suitable for comparison because it offers a 3D carbon network that has a much higher surface area than carbon cloth and carbon paper.

**Fig. 7:**
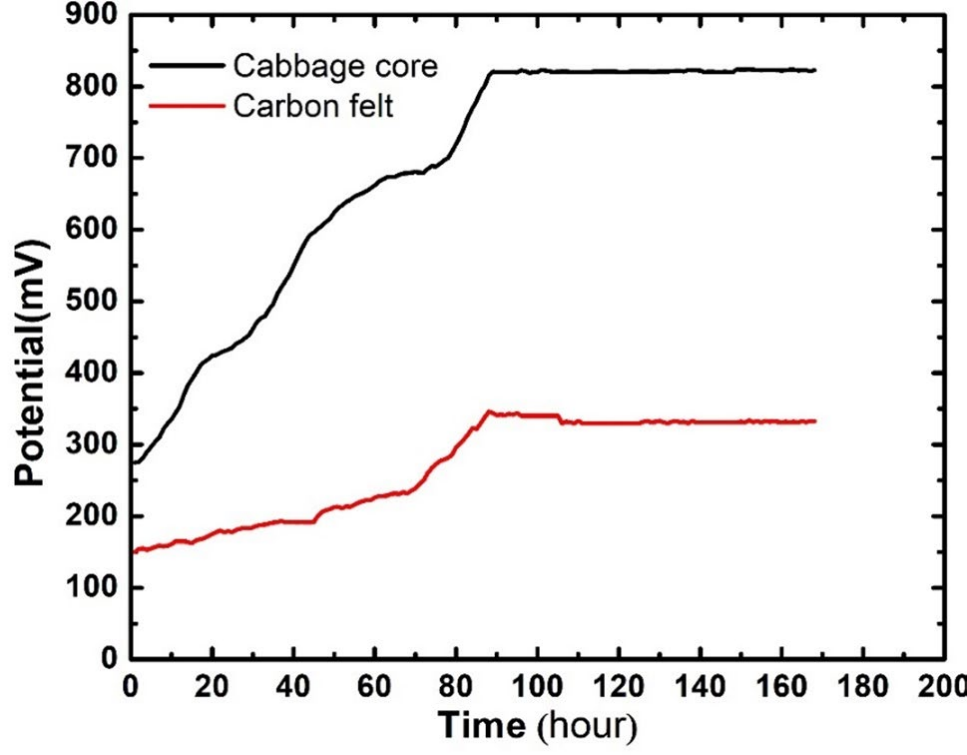
Output OCV of assembled cells

Open-circuit voltage (OCV) is one of the most important electrochemical measurements for evaluating the performance of microbial fuel cells. Figure 7 shows the OCV values for the cell assembled with the cabbage core waste anode and the control cell in which carbon felt was used as the anode. Both cells exhibit similar behavior, starting with a low open-circuit voltage (OCV) and gradually building up to reach their maximum value on the 4th day of operation. However, the cabbage core anode demonstrated a higher initial open-circuit voltage (OCV) of 275 mV and a significantly higher rate of increase in OCV values during the first 4 days of operation, achieving a maximum OCV value of 822.4 ± 5.2 mV, which outperformed the carbon felt anode by 470 ± 2.3 mV. This increase in OCV values can be attributed to the improvement of extracellular electron transfer in the cabbage core waste anode. Moreover, the rough and porous surface of the cabbage core anode increased the biodiversity of microorganisms on its surface, which plays an important role in pollutant oxidation and enhances the electrochemical properties of the biofilm [38].

**Fig. 8:**
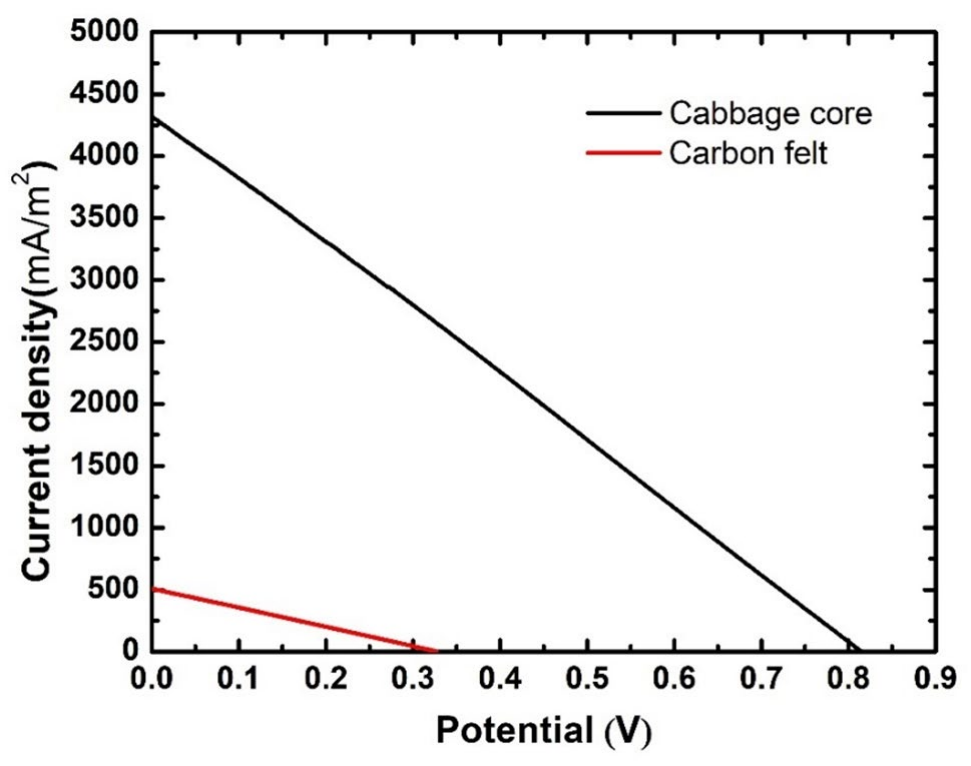
Polarization curves for assembled cells

**Fig. 9:**
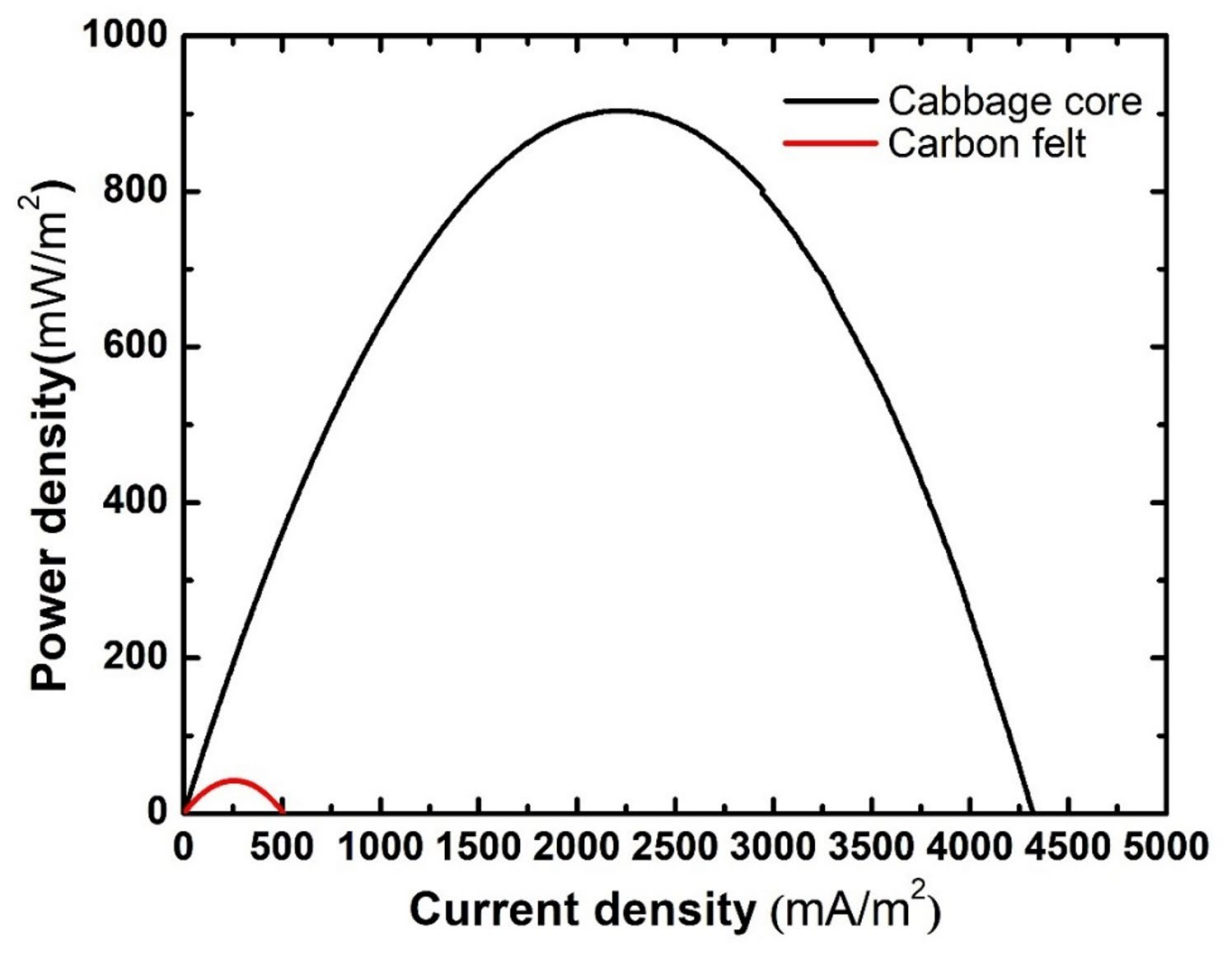
Maximum power density curves for assembled cells

Another term to evaluate the performance of microbial fuel cells is power generation and polarization curves. The voltage output values for the assembled microbial fuel cells and their corresponding current density are shown in Fig. 8. It’s obvious that the cabbage core anode generated much higher current density values compared to the carbon felt, with a maximum current density of 4321.67 mA/m², which is 8.5 times the maximum current density generated by the carbon felt anode. Subsequently, the cabbage core anode superiorly outperformed the carbon felt anode in terms of power generation. Figure 9 shows power density values for both used anodes, with the cabbage core anode having a maximum power density of 904.1 mW/m², which is 21.5 times the power density obtained from the carbon felt anode (42.1 mW/m²). This significant increase in both power density and current density can be attributed to the high surface area of the cabbage core anode, which allowed a much higher amount of electro active bacteria to attach to the surface of the anode and also the rough surface of the anode with so many macro and micro pores that made it favorable for microorganisms to form their biofilm on the surface of the anode.

**Fig. 10:**
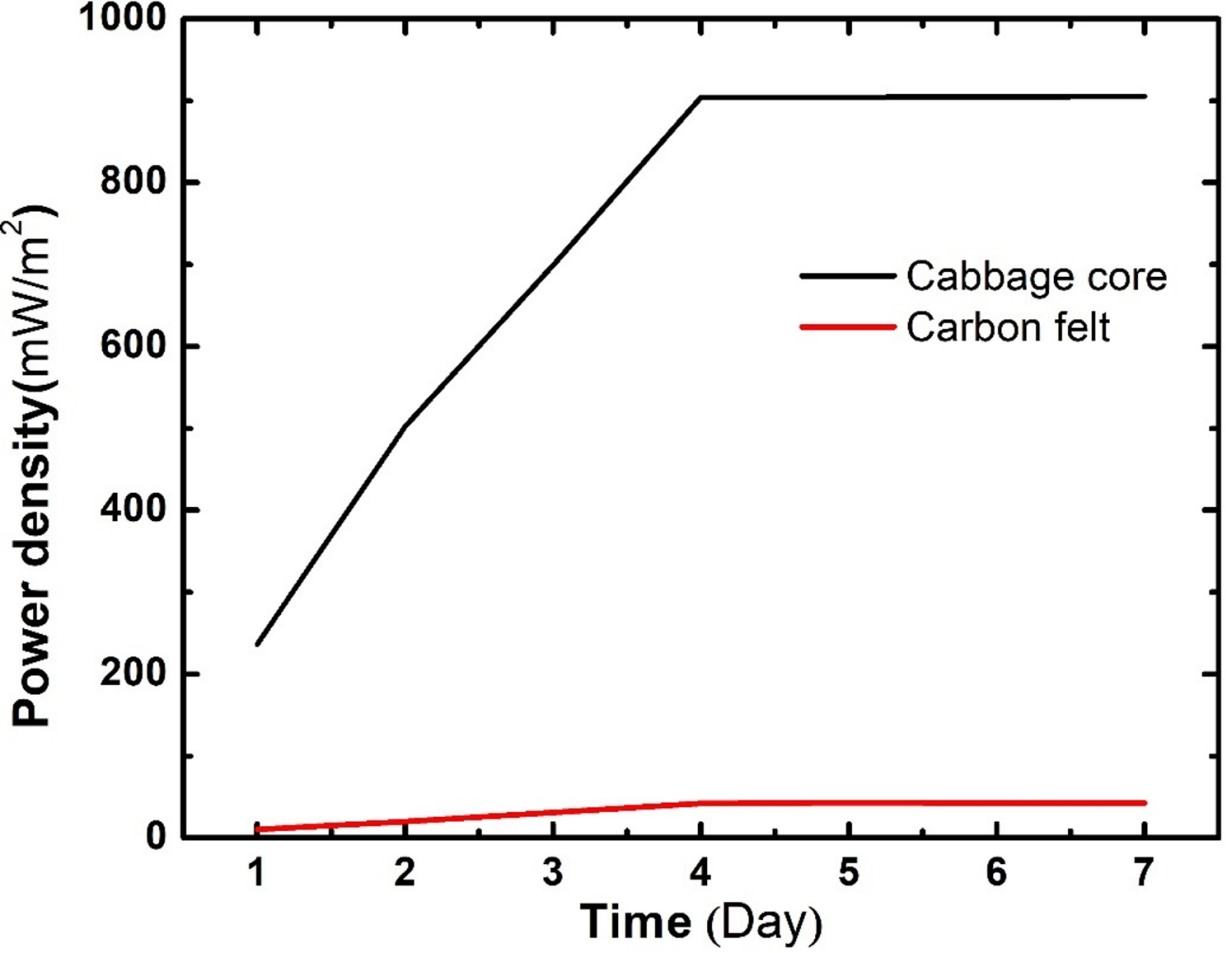
Power density increase over time

The power generation profile shown in Fig. 10 shows the gradual increase in maximum power generation for both cells assembled with a cabbage core anode and carbon felt. It’s obvious that the cabbage core anode had a significantly higher power generation value at the beginning of cell operation and maintained a very high rate of increase in power generation for the first 4 days of operation until reaching a maximum power density value of 904.1 mW/m² on the fourth day of operation. On the other hand, the carbon felt anode demonstrated a very low rate increase in power generation over time and stopped increasing on the third day. The wide gap between the power density values of the assembled cells can be attributed to the structure of the cabbage core anode, which provides electroactive microorganisms with a very large surface area and numerous micro- and macro-pores that are favorable for biofilm formation. Additionally, the presence of graphitic domains within the carbon atom structure improved extracellular electron transfer, which significantly increased OCV values and power density.

Cyclic voltammetry provides two important insights about the proposed electrode. First, the presence of any compounds that can be oxidized within the anode structure can be inferred from the presence of redox peaks in the cyclic voltammogram. Second, estimating the specific capacitance of the anode. Second, estimating the specific capacitance of the anode. Specific capacitance can be described as the area of the cyclic voltammogram.

**Fig. 11:**
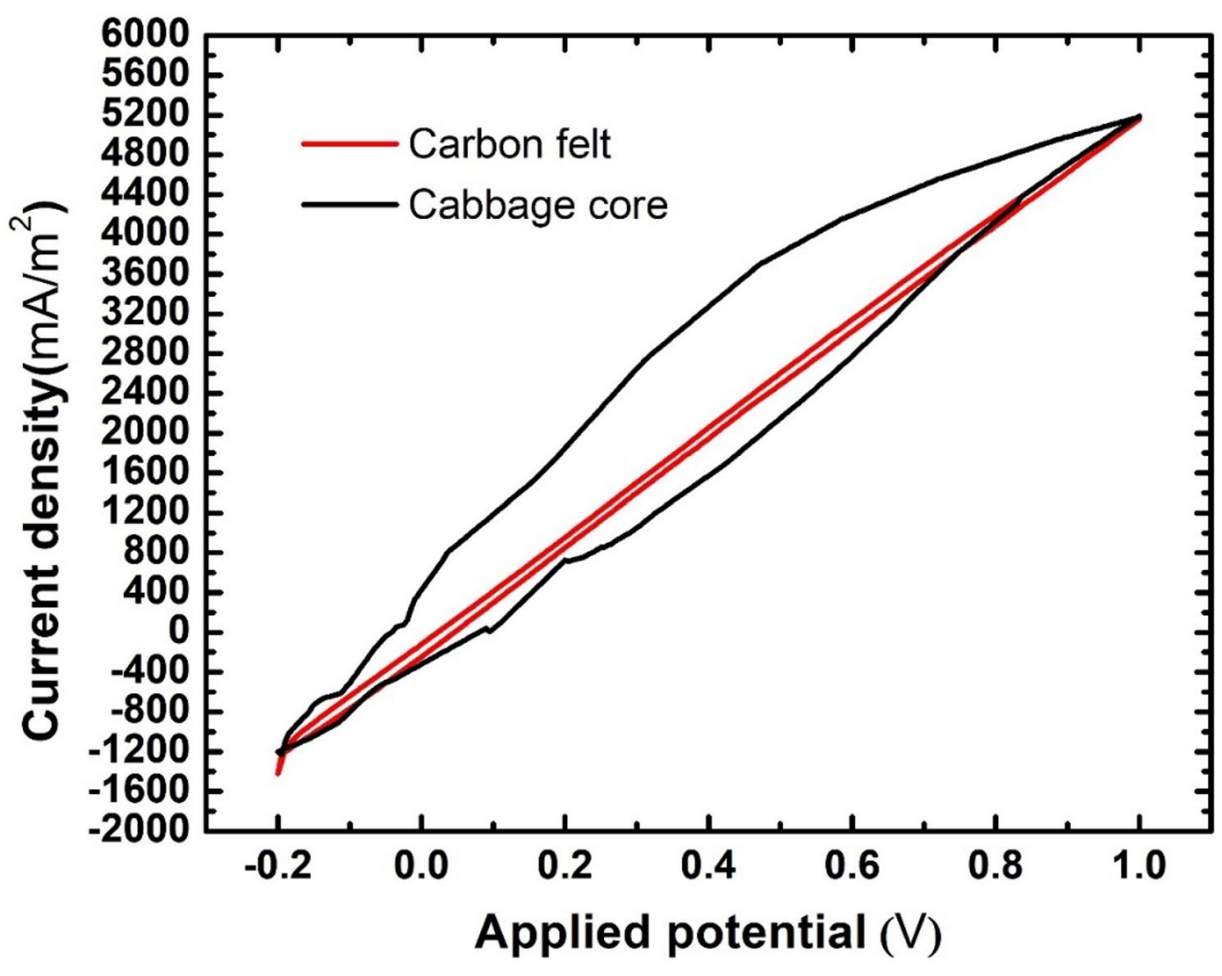
Cyclic voltammetry for MFC assembled with cabbage core anode

Materials with high specific capacitance have a good ability to absorb ionic and partially ionic compounds. Figure 11 shows the cyclic voltammogram for the cabbage core anode using a scan rate of 1 mV/s. It’s obvious that the structure of the anode is clear from any oxidizable compounds due to the absence of redox peaks. Moreover, it’s evident from the difference in voltammogram area that the carbonized cabbage core anode had a significantly higher specific capacitance than the carbon felt anode. This indicates that the carbonized cabbage core anode has a higher electrosorption capacity toward charged particles in wastewater, which is favorable for microbial fuel cell operation.

Cyclic voltammetry measurements were conducted on the plain (abiotic) cabbage core-derived anode prior to microbial inoculation to evaluate the presence of any intrinsic redox-active species or electrochemically oxidizable surface functionalities. The absence of distinct redox peaks confirms that the anode does not exhibit inherent Faradaic activity and that the observed current generation during MFC operation is not influenced by electrode-derived redox reactions. This finding supports that electricity production in the assembled microbial fuel cells arises predominantly from biologically mediated processes following biofilm formation, rather than from the electrochemical activity of the anode material itself.

### COD removal and microbial community analysis

The removal of COD is another way to compare the reference electrode with the changed electrode. The rate of chemical oxygen demand (COD) elimination is a measure of how well microbes break down biodegradable contaminants in wastewater. Each cell was sampled after 7 days, and the absorbance value was measured with a spectrophotometer after digestion. The COD concentration was then calculated for the MFCs’ influent and effluent by utilizing a standard curve. At the same time, every cell was subjected to the identical circumstances. As shown in Table 1, the initial COD and BOD values of the wastewater sample are significantly higher than the normal values for COD and BOD in municipal wastewater or wastewater from other industries. The COD value for the effluent of the cell assembled with the cabbage core anode was 29,640 mg/L, corresponding to a COD removal ratio of 58.8%.

On the other hand, the COD value for the effluent of the carbon felt cell was 35,821 mg/l with a COD removal ratio of 50.2%. Despite being able to oxidize big amounts of organic pollutants, microbial fuel cells couldn’t produce effluents that meet environmental regulatory demands for safe wastewater disposal. This confirms the fact that microbial fuel cells can’t be considered a stand-alone wastewater treatment system.

### The effect of changing the type of wastewater

**Fig. 12:**
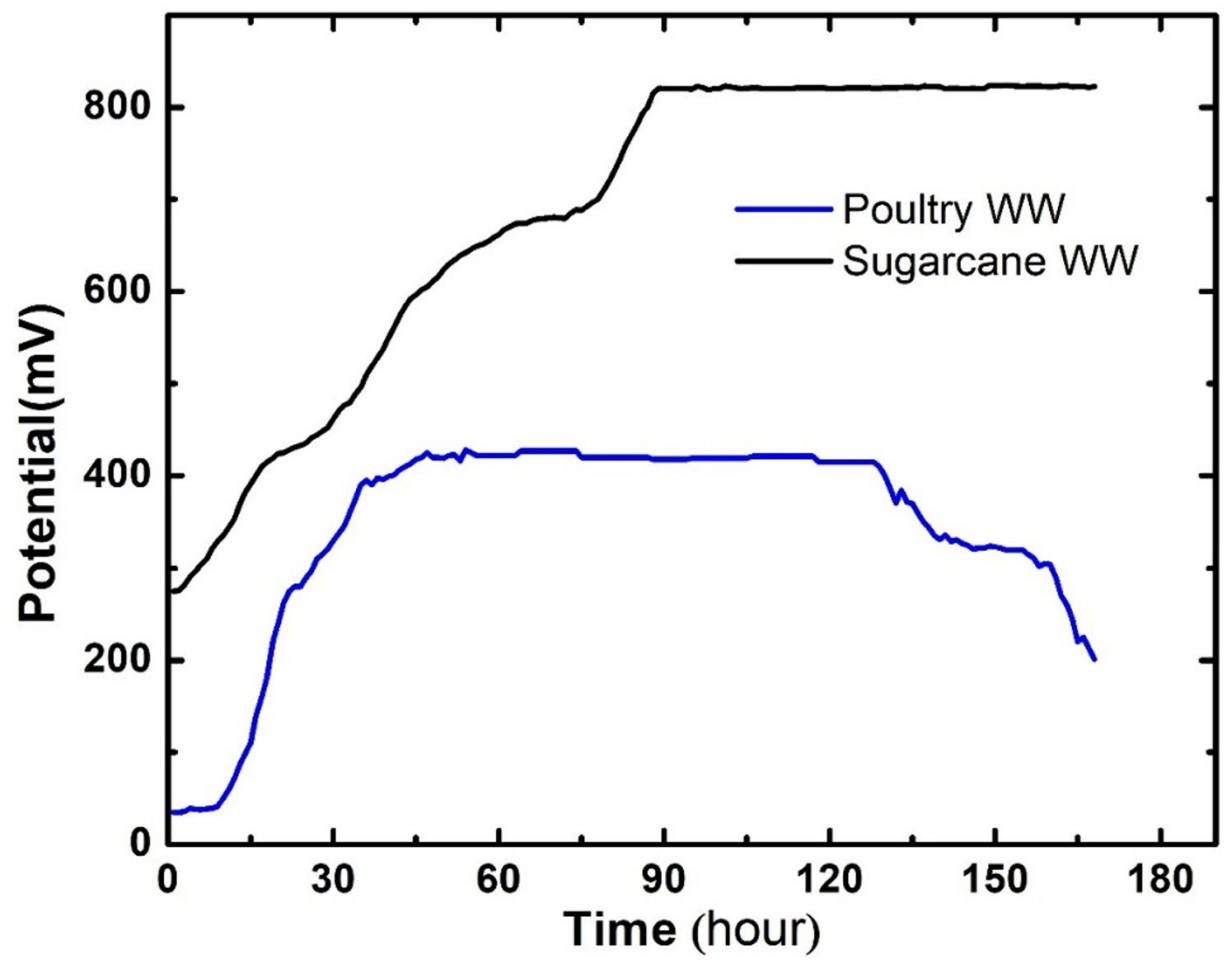
Open circuit voltage of operated cells with different wastewater

Two types of wastewater were used in this study, sugarcane wastewater and poultry wastewater, to explore the effect of changing the type of wastewater on the power generation efficiency of the microbial fuel cell. Results show that cells assembled with sugarcane wastewater outperformed cells assembled with poultry wastewater in both operation stability and power density values. Figure 12 shows the open-circuit voltage (OCV) of cells assembled with both types of wastewater using a carbonized cabbage core as the anode. Both cells started at the same open-circuit voltage (OCV) value and demonstrated similar values over the first day of operation. However, the poultry wastewater cell stabilized at 409.82 ± 1.2 mV and then started declining on the fourth day.

**Fig. 13:**
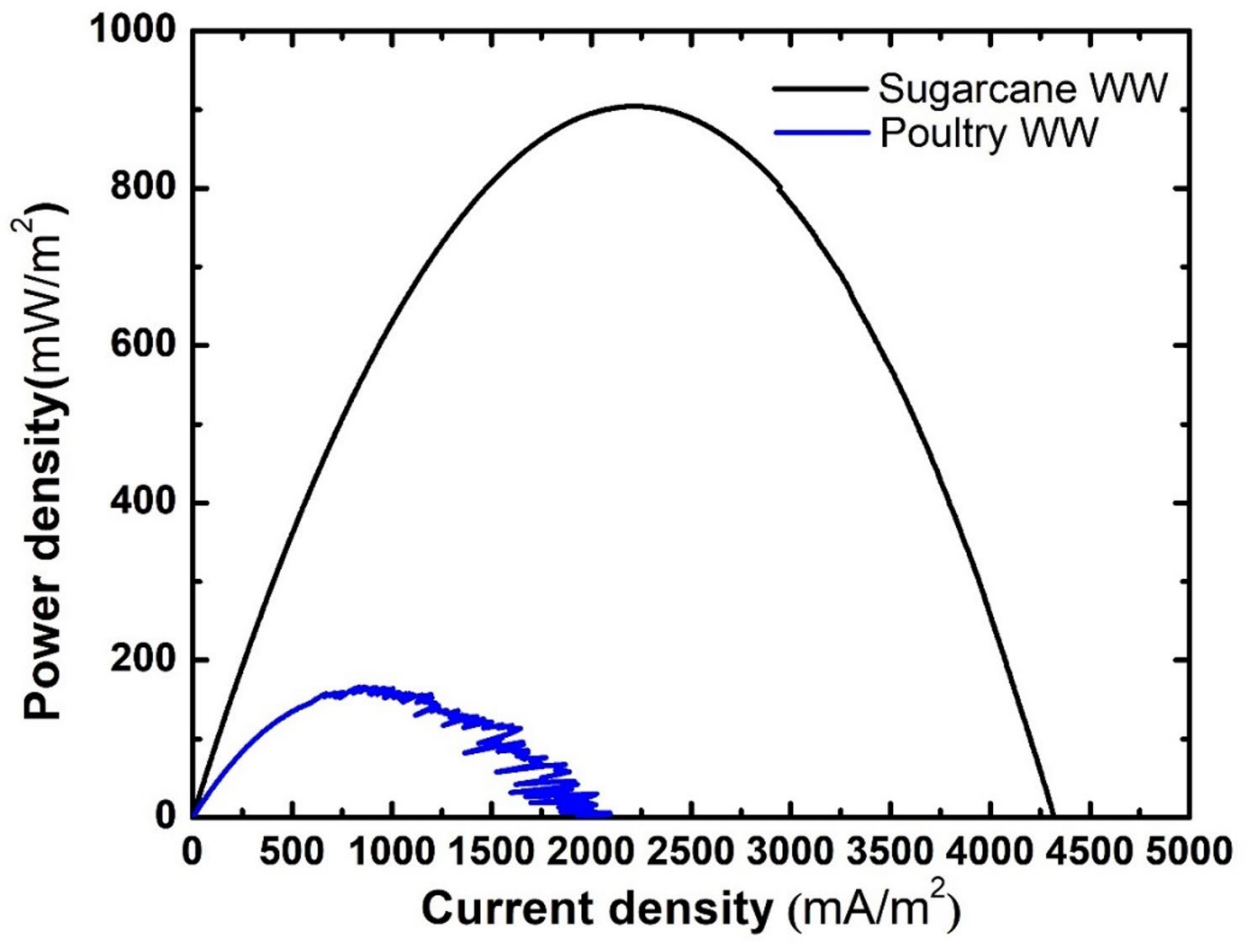
Power density generated by cells operated with different types of wastewater

As for power density (Fig. 13), Poultry wastewater demonstrated very low power generation, with a maximum power density of 158.01 ± 2 mW/m² and significant fluctuation in current density values. The poor performance of the poultry wastewater cell can be attributed to the differences in the biodegradability of organic pollutants and the differences in microbial communities in both cells, as well as the unfavorable electrochemical properties of poultry wastewater when used as an anolyte. These results highlight the importance of choosing a suitable type of wastewater for microbial fuel cell operation.

### Bacterial analysis

**Fig. 14:**
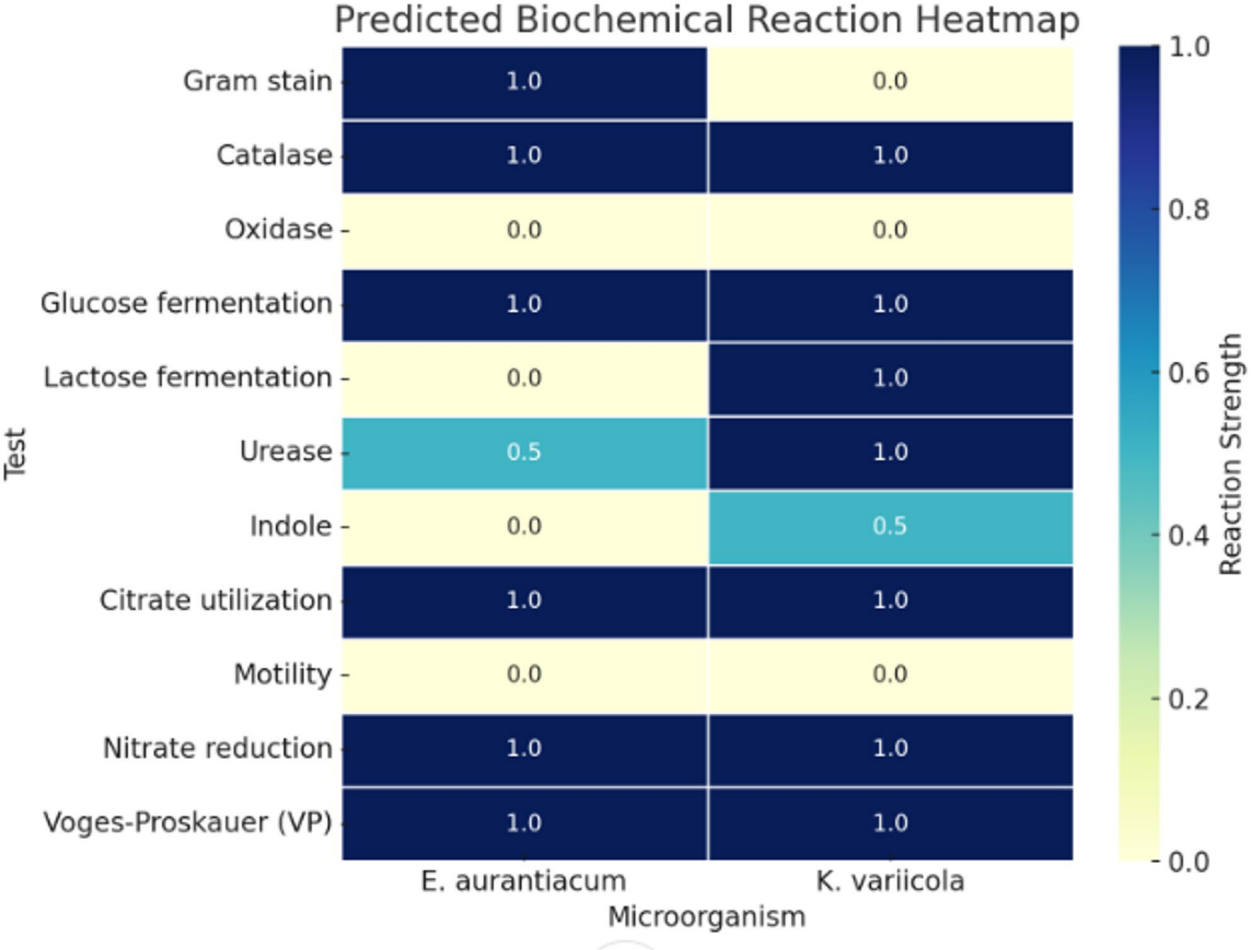
Heatmap of Predicted Biochemical Reaction Profiles for *E. aurantiacum* and *K. variicola* using VITEK 2 Knowledge Base (https://www.biomerieux.com/). This heatmap illustrates the predicted outcomes of key biochemical tests performed on *Exiguobacterium aurantiacum* and *Klebsiella variicola*. The color gradient represents the reaction strength, where 1.0 indicates a positive result, 0.0 indicates a negative result, and 0.5 denotes a variable or strain-dependent reaction. Biochemical tests include Gram staining, catalase, oxidase, glucose and lactose fermentation, urease, indole, citrate utilization, motility, nitrate reduction, and Voges-Proskauer (VP) test

Microorganism identification was performed using the bioMérieux VITEK MS system. The analysis of Sample (S4) yielded a high-confidence identification of *Exiguobacterium aurantiacum* with a 99.9% probability score. Meanwhile, sample (S1) was identified as *Klebsiella variicola*. To further characterize the isolates, predicted biochemical profiles were compiled based on known characteristics of the identified species. A heatmap summarizing the expected outcomes of eleven key biochemical tests is presented in Fig. 14. *E. aurantiacum* demonstrated positive reactions for Gram staining, catalase, glucose fermentation, citrate utilization, nitrate reduction, and Voges-Proskauer test, while tests for oxidase, lactose fermentation, indole, and motility were negative. Urease activity was noted to be variable among strains.

*K. variicola* showed positive reactions for catalase, glucose and lactose fermentation, urease, citrate utilization, nitrate reduction, and Voges-Proskauer test, with negative reactions for oxidase, indole, and motility. Indole production was variable in some strains, as reported in previous literature. These biochemical predictions align with the known phenotypic profiles of both species and provide additional support to the VITEK MS identification results.

**Fig. 15:**
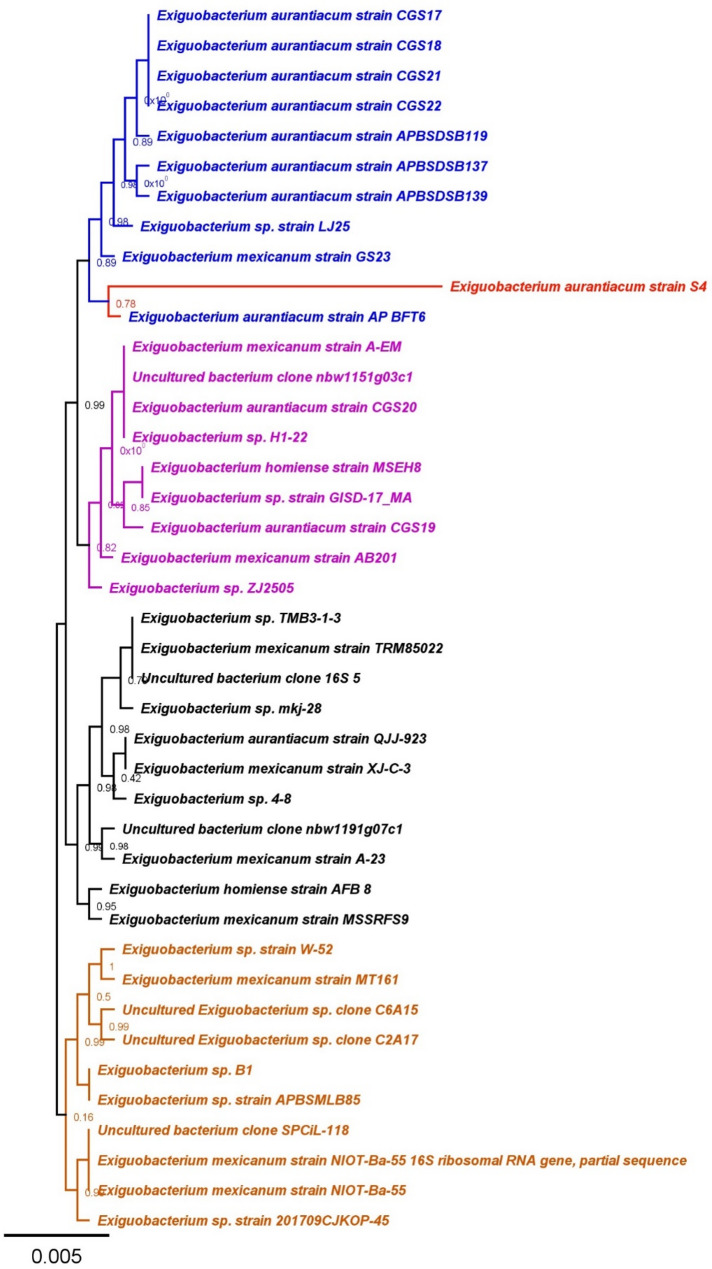
Phylogenetic tree of *Exiguobacterium* strains based on 16 S rRNA gene sequences. A phylogenetic tree was constructed to analyze the evolutionary relationships among different *Exiguobacterium* strains using 16 S rRNA gene sequences. The tree includes multiple strains of *Exiguobacterium aurantiacum*, *Exiguobacterium mexicanum*, and other *Exiguobacterium* species, along with uncultured bacterial clones. Different clades are color-coded to distinguish major groupings. Branch support values (posterior probabilities) are indicated at the nodes, with values closer to 1 signifying stronger support. The scale bar represents genetic distance (0.005), illustrating sequence divergence. Our strain is highlighted in red

The phylogenetic tree (Fig. 15) illustrates the evolutionary relationships among various *Exiguobacterium* strains based on 16 S rRNA gene sequences. The *Exiguobacterium aurantiacum* strains form distinct clusters, primarily in blue and red, indicating their genetic similarity. The phylogenetic placement of strain S4 (in red) within a well-defined clade suggests that it shares a common ancestor with other *Exiguobacterium* species, especially *Exiguobacterium aurantiacum* strain AP BFT6, indicating an evolutionary link. Whereas *Exiguobacterium mexicanum* strains are more dispersed across multiple clades, reflecting greater genetic diversity. Some uncultured *Exiguobacterium* clones appear closely related to known strains, suggesting potential distinct species. Branch support values indicate strong confidence in most relationships, particularly where values approach 1, though some branches show lower support, indicating uncertainty. Notably, *Exiguobacterium aurantiacum* strain S4 emerges as an early diverging member in its subgroup. Several *Exiguobacterium* sp. strains are interspersed among well-defined species, reinforcing the possibility of uncharacterized diversity within the genus. The genetic divergence scale (0.005) highlights the subtle sequence variations that contribute to species differentiation.

**Fig. 16:**
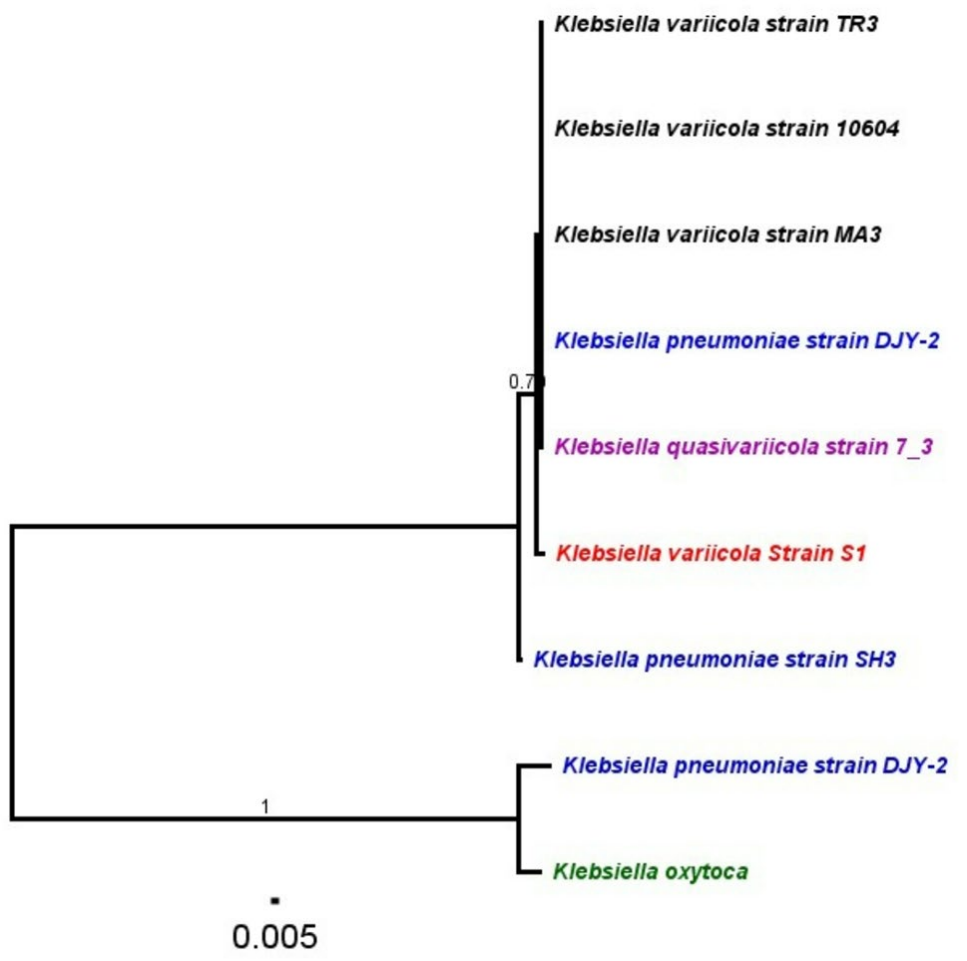
Phylogenetic tree of *Klebsiella* strains based on 16 S rRNA gene sequences. A phylogenetic tree was constructed to analyze the evolutionary relationships among different Klebsiella strains using 16 S rRNA gene sequences. The tree includes multiple strains of *Klebsiella variicola*, *Klebsiella pneumoniae*,* Klebsiella quasivariicola*, and *Klebsiella oxytoca*. Different clades are color-coded to distinguish major groupings. Branch support values (posterior probabilities) are indicated at the nodes, with values closer to 1 signifying stronger support. The scale bar represents genetic distance (0.005), illustrating sequence divergence. Our strain *K. variicola* strain S1 is highlighted in red colour

The phylogenetic tree (Fig. 16) revealed that the *Klebsiella variicola* strains (TR3, 10604, and MA3) clustered together within a well-supported clade. Notably, the *Klebsiella variicola* strain S1, isolated in the current study, grouped closely with *Klebsiella quasivariicola* strain 7_3 and *Klebsiella pneumoniae* strain DJY-2, forming a distinct subclade from other *K. variicola* reference strains, as shown in Fig. 14. Interestingly, the *Klebsiella variicola* strain S1, isolated in the current study, emerged in a separate subclade, distinct from the other *K. variicola* strains. This placement indicates potential genetic divergence and highlights the genomic diversity within the *Klebsiella variicola* species complex.

Additionally, *Klebsiella pneumoniae* strains (DJY-2 and SH3) formed a separate cluster within the tree. *Klebsiella oxytoca*, included as an outgroup, was positioned distantly from the other *Klebsiella* species, confirming its phylogenetic distinction within the genus.

Branch support values (posterior probabilities) were generally high, particularly for the major clades, indicating robust phylogenetic relationships. The scale bar represents a genetic distance of 0.005 substitutions per site, reflecting sequence divergence across the included strains.

The present study utilized the bioMérieux VITEK MS system for rapid and accurate identification of bacterial isolates obtained from the tested samples. The system confidently identified *Exiguobacterium aurantiacum* and *Klebsiella variicola*, with a 99.9% probability score for *E. aurantiacum. Exiguobacterium aurantiacum* is a Gram-positive, facultatively anaerobic bacterium often isolated from diverse environments, including water, soil, and food products. While traditionally considered a low-pathogenicity organism, it has been occasionally reported in clinical settings, particularly among immunocompromised patients. The predicted biochemical profile of the isolate in this study closely matched previously published descriptions, with positive reactions for catalase, glucose fermentation, nitrate reduction, and variable urease activity, reflecting the strain-dependent biochemical variability reported in the genus [39].

On the other hand, *Klebsiella variicola*—a member of the *Klebsiella* pneumoniae complex—is increasingly recognized as an emerging opportunistic pathogen. Historically misidentified as K. pneumoniae, this species has been implicated in a variety of clinical infections, including bloodstream infections, urinary tract infections, and pneumonia. The biochemical profile predicted for *K. variicola* in this study was consistent with its known phenotypic characteristics, including positive reactions for lactose and glucose fermentation, urease, citrate utilization, and Voges-Proskauer test [40]. Notably, the negative indole and oxidase reactions helped distinguish it from other members of the complex. The biochemical profiling provides a robust approach for accurately characterizing bacterial isolates, particularly when dealing with closely related species or atypical strains. The concordance between the VITEK MS identification and the predicted biochemical traits in this study highlights the value of combining molecular and phenotypic data for comprehensive microorganism characterization.

In conclusion, this study demonstrates the efficacy of VITEK MS for bacterial identification, supplemented by biochemical reaction profiles that provide additional phenotypic context. The reliable identification of *E. aurantiacum* and *K. variicola* contributes to the growing recognition of their potential environmental presence. The phylogenetic analysis of *Exiguobacterium* strains isolated from anode oxidation systems provides valuable insights into their evolutionary relationships and potential functional roles in electricity generation. The distinct clustering pattern of *Exiguobacterium aurantiacum* S4 suggests genetic divergence due to species-specific adaptations, possibly related to their electroactive capabilities in microbial fuel cells (MFCs).

Several uncultured *Exiguobacterium* clones closely related to known species suggest the presence of novel or previously uncharacterized strains with potential electrochemical activity. High branch support values indicate strong confidence in most major clades, particularly those with posterior probabilities close to 1. However, lower support values in some branches may reflect genetic recombination, horizontal gene transfer, or limitations in 16 S rRNA resolution, all of which are common in bacteria involved in dynamic environments such as bioelectrochemical systems.

The phylogenetic analysis highlights the evolutionary placement of *Exiguobacterium aurantiacum* strain S4, which forms a distinct cluster with *Exiguobacterium aurantiacum* strain AP BFT6, indicating a close genetic relationship. The relatively strong branch support suggests a reliable phylogenetic positioning, reinforcing its classification within the *E. aurantiacum* group. However, its divergence from other *E. aurantiacum* strains, such as CGS17 and CGS18, suggests potential genetic variations that may influence its metabolic capabilities, particularly its role in anode oxidation and electricity generation.

The isolation of strain S4 from an anode oxidation environment implies its ability to participate in extracellular electron transfer (EET), a key mechanism in microbial electricity production. The presence of closely related strains in different clades suggests possible adaptations that could enhance their electroactive potential. Future comparative genomic analyses between strain S4 and other Exiguobacterium strains may reveal specific genes or metabolic pathways involved in EET, such as cytochromes, pili formation, or redox-active metabolites. *Exiguobacterium* are low G+C, Gram-positive, facultative anaerobes that belong to the class Bacilli in the phylum Firmicutes [39]. It is a metabolically versatile genus with significant potential in microbial fuel cell (MFC) applications. Its ability to thrive in diverse and extreme environments, including high-salinity and temperature-fluctuating conditions, makes it a robust candidate for bioelectricity generation. Several *Exiguobacterium* species exhibit electrogenic properties, enabling them to transfer electrons to an electrode through extracellular electron transfer (EET) mechanisms. This enhances the efficiency of MFCs by improving power output and current generation.

Additionally, *Exiguobacterium* contributes to the degradation of organic matter in wastewater, making it valuable for bioenergy production and bioremediation in microbial fuel cell (MFC) systems [41]. Due to its adaptability and biofilm-forming capabilities, *Exiguobacterium* strain S4 can sustain stable power generation in MFCs, even under harsh conditions. Its ability to couple energy metabolism with electron transfer pathways highlights its potential for enhancing MFC performance, particularly in wastewater treatment and sustainable energy applications. The discovery of *Exiguobacterium* from an Egyptian source as a novel microbial fuel cell (MFC) biocatalyst expands the known geographical and functional diversity of this genus. Its ability to sustain power generation under varying environmental conditions highlights its potential for future applications in sustainable energy.

The phylogenetic analysis conducted in this study offers critical insights into the genetic relationships within the *Klebsiella* genus, particularly through 16 S rRNA gene sequencing. Consistent with prior research, reference strains of *Klebsiella variicola* clustered within a well-supported clade, reaffirming the genetic coherence typically attributed to this species [42]. However, the emergence of *K. variicola* strain S1 in a distinct subclade—closer to *K. quasivariicola* and *K. pneumoniae* than to other *K. variicola* strains—suggests a notable degree of intra-species diversity. This divergence may be attributed to unique genomic adaptations or historical horizontal gene transfer events, which are known to contribute to the evolutionary plasticity of *Klebsiella* species. Such findings challenge the traditional view of *K. variicola* as a genetically uniform species, underscoring the broader genomic diversity that may exist, particularly among environmental or non-clinical isolates. This diversity is especially relevant in the context of emerging applications, such as microbial fuel cells, where strain-specific metabolic traits can significantly influence performance.

The identification of strain S1, with its distinct phylogenetic profile, highlights the importance of continued genomic surveillance. Understanding the evolutionary dynamics of such strains for harnessing their biotechnological potential in sustainable energy systems. The Rationale for extracellular electron transfer by *Exiguobacterium aurantiacum* is genus-level traits of *Exiguobacterium*, which provide a coherent mechanistic basis for anodic extracellular electron transfer (EET) in our sugarcane-wastewater MFCs. First, *Exiguobacterium* spp. are strong biofilm formers that produce abundant extracellular polymeric substances (EPS), promoting intimate and persistent attachment to conductive surfaces and creating redox-active matrices favorable for electron exchange [43, 44]. Second, comparative genomics and recent phenotypic studies highlight exceptional metabolic versatility, broad transporter repertoires, and resilience to pH, salinity, UV, and metal stress, attributes that support robust respiration under the fluctuating, high-strength conditions typical of agro-industrial effluents [45, 46]. Third, members of the genus encode and secrete stress-tolerant redox enzymes/oxidoreductases that can catalyze key steps in outward EET, either directly to the anode or via secreted factors [47, 48]. Fourth, mixed-community electrogenesis in MFCs is often mediated and enhanced by quinone/flavin electron shuttles that simultaneously increase biofilm biomass and current output; such shuttle-compatible physiology provides a plausible route for *E. aurantiacum* to participate in mediated EET within the anode consortium [49, 50]. Finally, prior MFC studies on sugar-industry effluents consistently report Firmicutes-rich electroactive communities with strong power densities, aligning with the ecological placement of *E. aurantiacum* as a credible contributor to the current generation in this wastewater matrix [51].

*Klebsiella variicola* is a plausible contributor to anodic extracellular electron transfer (EET) in MFCs for four complementary reasons. First, biofilm-mediated electron exchange is feasible: wild-type *K. variicola* adheres to carbon electrodes, reduces charge-transfer resistance, and boosts power output during wastewater operation, indicating a functional anode biofilm capable of electron exchange [52]. Second, mediated EET via self-produced quinone shuttles has been demonstrated in the genus *Klebsiella pneumoniae* L17 secretes 2,6-di-tert-butyl-p-benzoquinone that ferries electrons from cells to the anode even when direct contact is limited, supporting a conserved shuttle-based mechanism relevant to *K. variicola* [53]. Third, a facultative Klebsiella strain closely related to *K. variicola* (SQ-1) forms multilayer electrode biofilms, generates high current densities, and couples respiration to external acceptors (e.g., hydrous ferric oxide), demonstrating outer-electron respiration consistent with EET [54]. Finally, under electrode-respiring conditions, *Klebsiella* shifts central metabolism toward anaerobic respiration with electrons exported to the anode, confirming integration of electrode-based electron disposal into its metabolic network [55]. Together, these lines of evidence support *K. variicola* as a credible electrogen in mixed anode consortia treating agro-industrial effluents. Future work will include high-resolution SEM imaging combined with biofilm-specific staining and advanced microscopy techniques to study biofilm architecture of *Exiguobacterium aurantiacum* and *Klebsiella variicola*, extracellular polymeric substances (EPS), and cell–electrode interactions in detail.

## Conclusions

A sustainable and low-cost microbial fuel cell anode was fabricated by directly carbonizing the cabbage core stalk at a relatively low carbonization temperature of 800 °C. The proposed anode was evaluated in a double-chamber microbial fuel cell. It demonstrated a significant improvement in electrochemical performance compared to carbon felt despite the presence of difficulties in the electrode structure. The excellent electrochemical performance of the proposed anode can be attributed to the honeycomb-like surface morphology and the presence of graphitic domains within the anode structure. Moreover, a newly isolated electrogenic extremophilic bacteria in Egypt was found on the surface of the anode, which promotes the importance of discovering electrogenic bacterial strains that can sustain extreme conditions. The structure of the anode can be further modified by catalyzing the carbonization process using transition metals or doping with nitrogen.

## Supplementary Information

Below is the link to the electronic supplementary material.

Supplementary Material 1.

Supplementary Material 2

## Data Availability

• It is appropriate to request the datasets used and/or analyzed in this study from the corresponding author. • The data collected and analyzed for this investigation are all contained in this publication.
