# Supplementary material for: Upcycling agro-waste into sustainable bioenergy: using carbonized cabbage core wastes bio-anodes and novel bacterial insights in MFCs for sugarcane wastewater treatment
Source: Microb Cell Fact. 2026 Mar 2;25:86. doi: 10.1186/s12934-026-02954-7 (PMC13059587; doi:10.1186/s12934-026-02954-7)
Supplement: Supplementary file 1 — Supplementary Material 1. [file 12934_2026_2954_MOESM1_ESM.docx]

**Upcycling Agro-Waste into Sustainable Bioenergy: Using Carbonized Cabbage Core Wastes Bio-Anodes and Novel Bacterial Insights in MFCs for** **Sugarcane Wastewater Treatment**

**Ahmed Abotaleb^1^, Mai Ramadan^1^, Dina H. Amin^2^, Alaa F. Elsayed^2^, Nasser A. M. Barakat^3^, Amina Shaltout^1^, Abeer El Shahawy^1^***

^1^ Department of Civil Engineering, Faculty of Engineering, Suez Canal University, P.O. Box 41522, Ismailia, Egypt: ahmedelsayed12@eng.suez.edu.eg; mai.ramdan@eng.suez.edu.eg; amina_shaltout@eng.suez.edu.eg; [abeer_shahawi@eng.suez.edu.eg](mailto:abeer_shahawi@eng.suez.edu.eg).

^2^ Department of Microbiology, Faculty of Science, Ain Shams University, Cairo, 1566, Egypt, dina.hatem@sci.asu.edu.eg; alaafayez@sci.asu.edu.eg.

^3^Chemical Engineering Department, Faculty of Engineering, Minia University, Minya 61519, Egypt, nasbarakat@mu.edu.eg (N.A.M.B.).

*Corresponding author: [abeer_shahawi@eng.suez.edu.eg](mailto:abeer_shahawi@eng.suez.edu.eg)

**Supplementary**


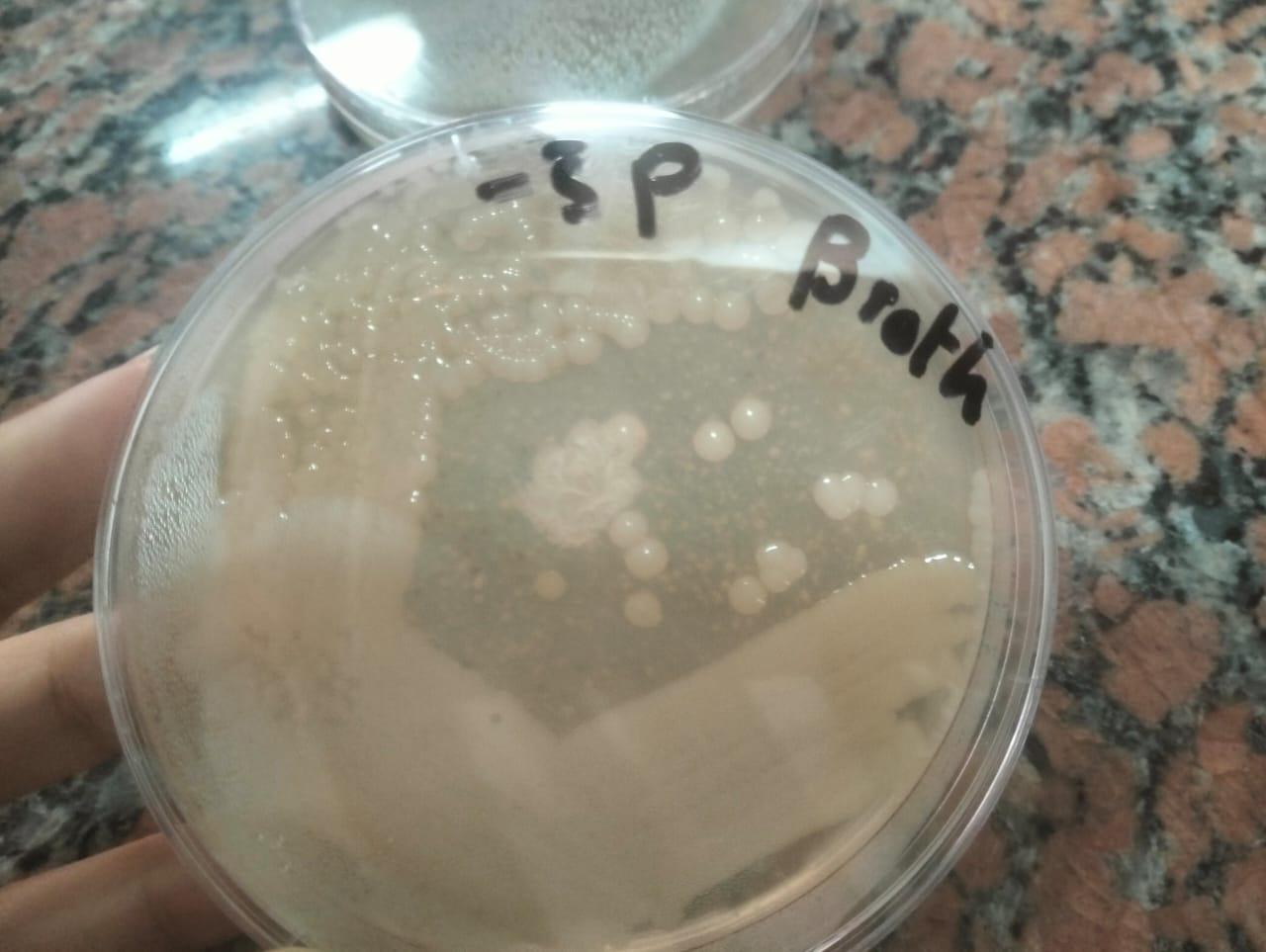


*Klebsiella variicola S1*


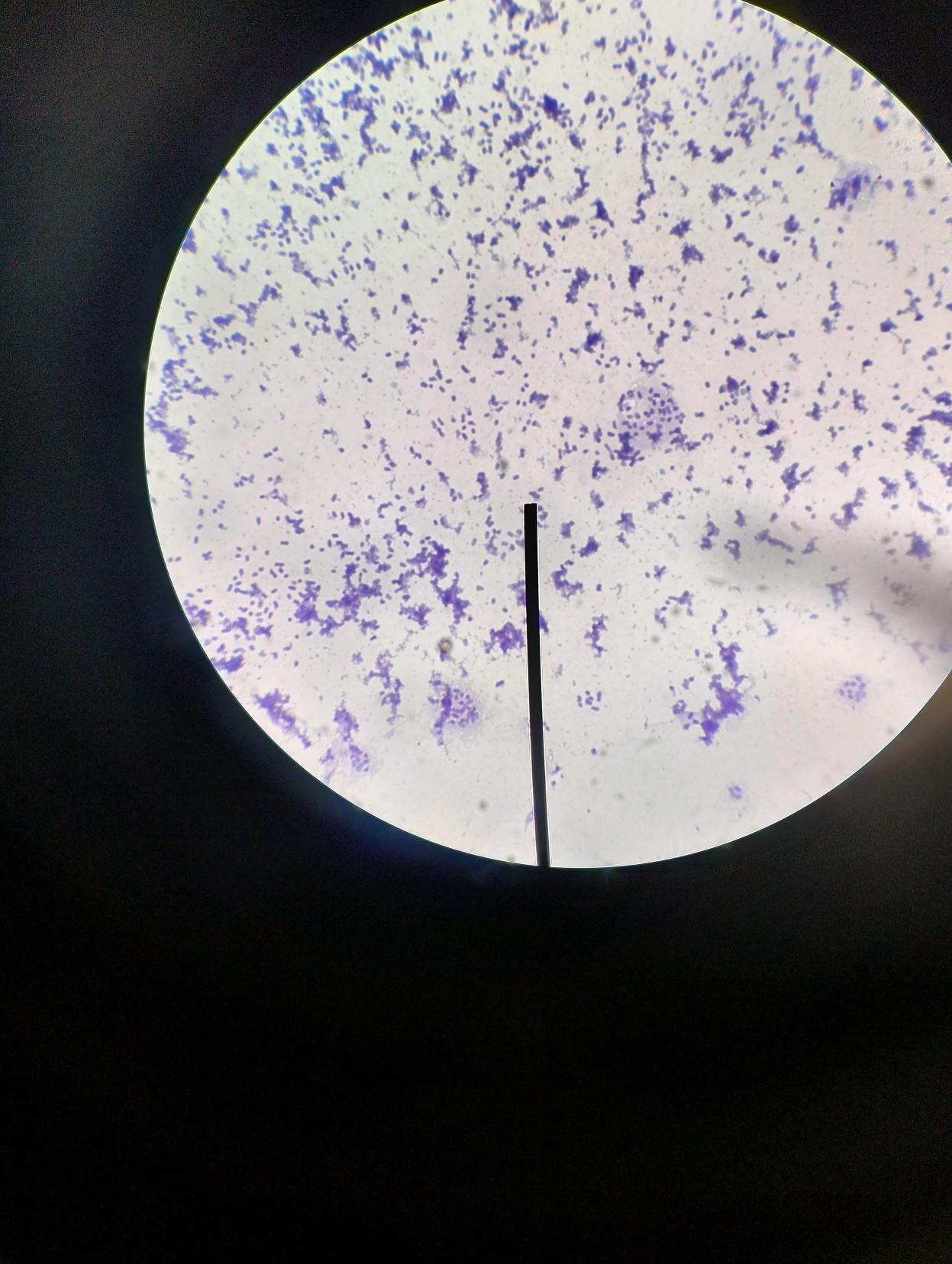


*Klebsiella variicola S1- Rod shaped Bacilli*


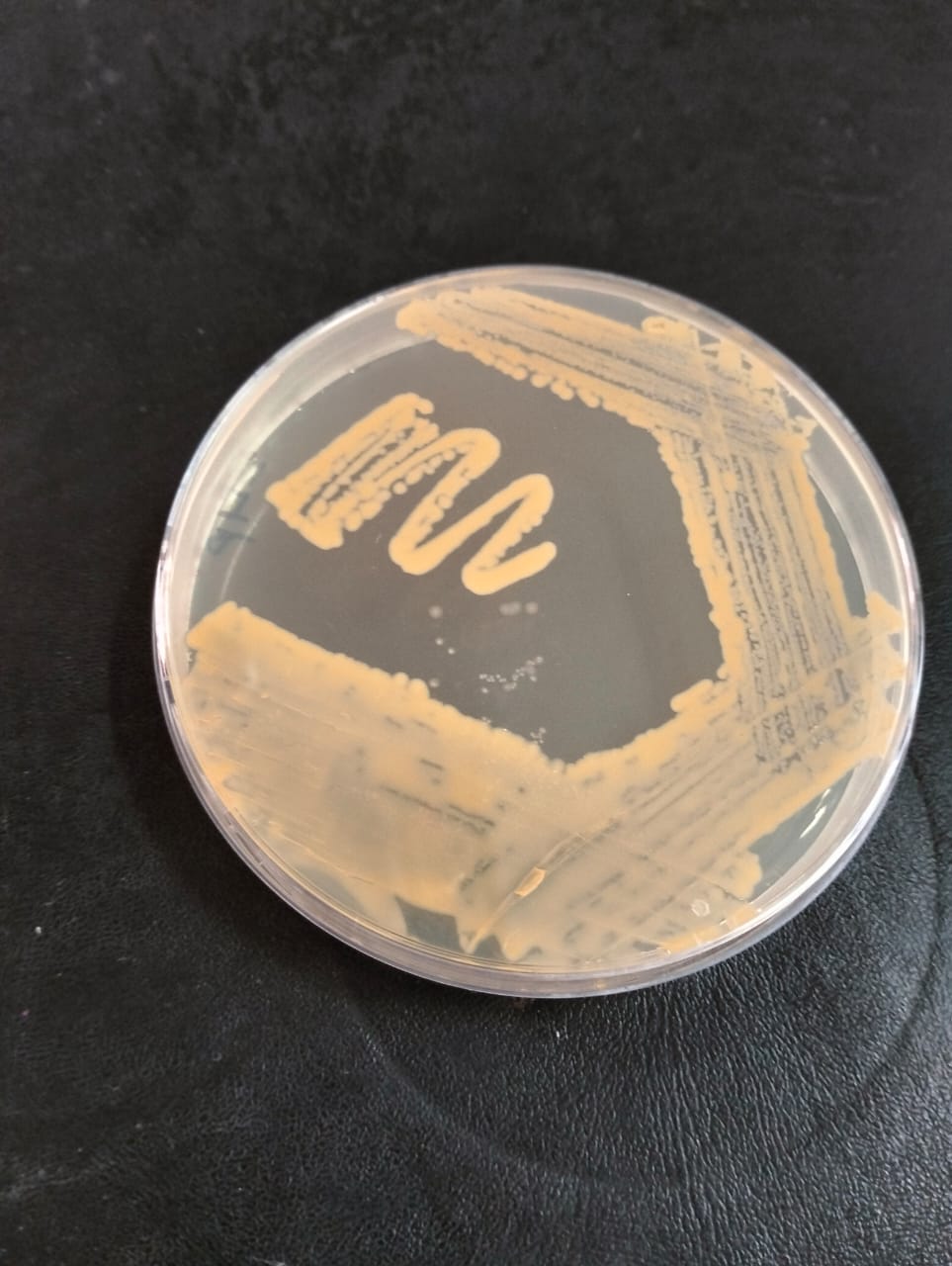


*Exiguobacterium aurantiacum, S4*


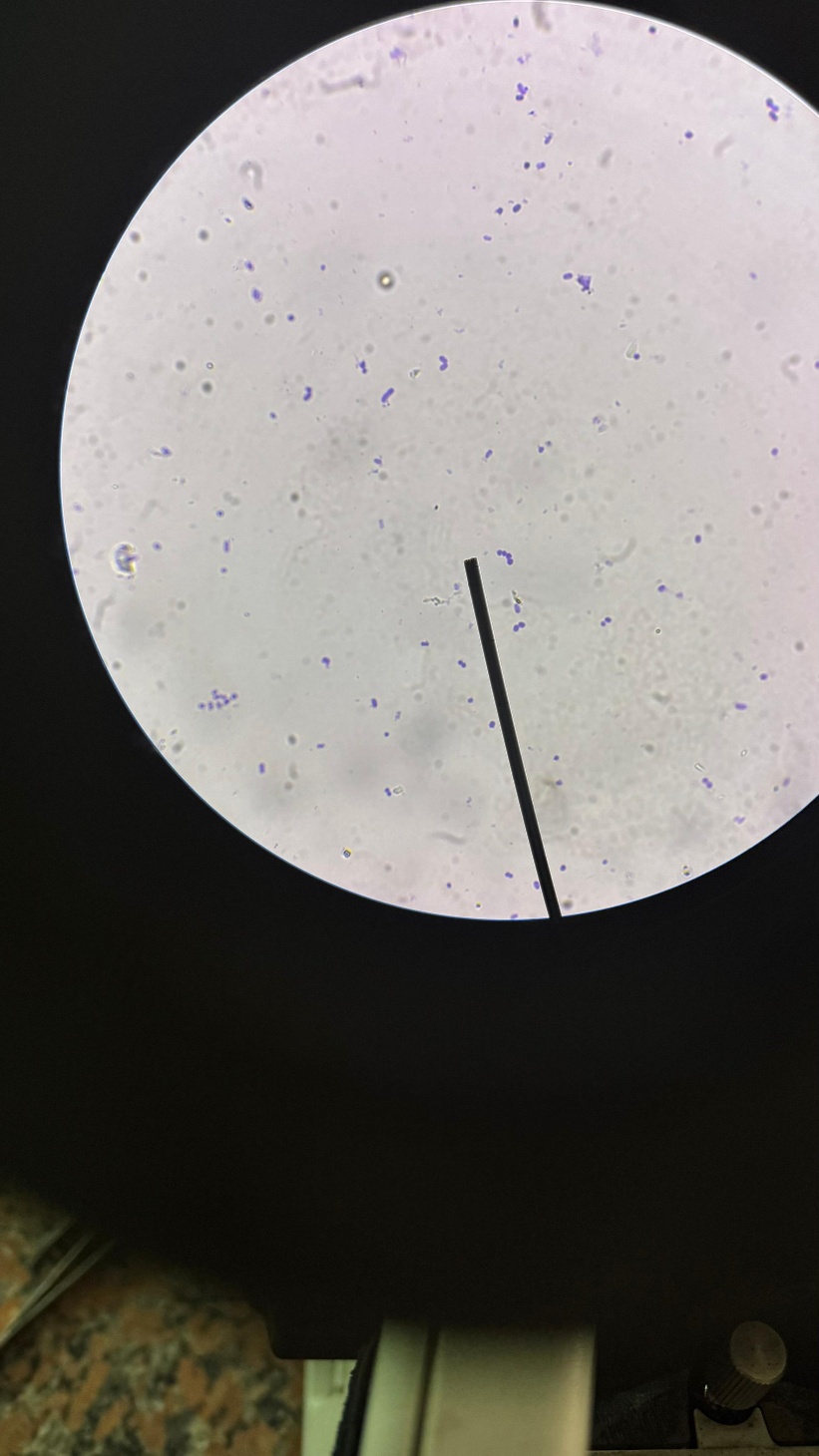


*Exiguobacterium aurantiacum, S4*

*Bacilli*

**Methods:**

**2.6. Bacterial analysis**

**2.6.1. Biochemical identification using VITEK**

Microorganism identification of the selected strains (S4-S1) was performed using the VITEK® MS system (bioMérieux, France) based on Matrix-Assisted Laser Desorption Ionization Time-of-Flight Mass Spectrometry (MALDI-TOF MS) (bioMérieux, 2025). Isolates were sub-cultured on appropriate agar media and incubated under optimal conditions. Freshly grown colonies were applied onto a VITEK® MS-DS target slide, overlaid with 1 μL of α-cyano-4-hydroxycinnamic acid (CHCA) matrix solution, and allowed to dry. The target slide was then loaded into the VITEK® MS instrument, and spectra were acquired using the manufacturer's settings. Identification was performed by comparing the obtained spectra against the VITEK MS database, following the CSEFRA-WI-29 equipment manual guidelines. The predicted biochemical reaction profiles for each strain were retrieved from the VITEK 2 Knowledge Base (bioMérieux, Marcy-l’Étoile, France), which provides algorithm-based predictions of metabolic and enzymatic activities derived from large-scale phenotypic testing data.

**2.6.2. Molecular identification of the selected bacterial strains**

**DNA Extraction**

Genomic DNA was extracted from bacterial samples (S1-S4) using the QIAamp DNA Mini Kit (QIAGEN, Germany), following the manufacturer's protocol. Briefly, 20 μL of QIAGEN protease was added to the bottom of a 1.5 mL microcentrifuge tube, followed by 200 μL of the sample. Then, 200 μL of Buffer AL was added and mixed by pulse vortexing for 15 seconds. The mixture was incubated at 56°C for 10 minutes. After a brief centrifugation to remove droplets from the lid, 200 μL of 96% ethanol was added and remixed by vortexing. The lysate was transferred to a QIAamp Mini spin column, placed in a 2 mL collection tube, and centrifuged at 8000 rpm for 1 minute. The column was then washed sequentially with 500 μL of Buffer AW1 and Buffer AW2, each followed by centrifugation. A final centrifugation step was performed to remove the residual wash buffer. DNA was eluted by adding 100 μL of Buffer AE to the column, incubating at room temperature for 1 minute, and centrifuging at 8000 rpm for 1 minute. The eluted DNA was stored at −20°C until further use.

**Preparation of PCR Master Mix**

Polymerase Chain Reaction (PCR) amplifications were performed using the Emerald Amp GT PCR Master Mix (2× premix) (Takara, Code No. RR310A) following the manufacturer's protocol. For each 25 μl reaction, the PCR mixture was prepared by combining 12.5 μl of the 2× premix with 4.5 μl of PCR-grade water. To this, 1 μl each of forward and reverse primers (20 pmol) was added, followed by 6 μl of template DNA. The final reaction volume was adjusted to 25 μl. The thermal cycling conditions for amplifying the target 16S rRNA gene were optimized based on the manufacturer's recommendations for Emerald Amp GT PCR Master Mix. The cycling protocol began with an initial denaturation step at 94°C for 5 minutes. This was followed by 35 cycles of denaturation at 94°C for 30 seconds, annealing at 56°C for 1 minute, and extension at 72°C for 1 minute. A final extension step was performed at 72°C for 10 minutes to ensure complete amplification of the target DNA fragments. A DNA molecular weight marker was prepared by gently mixing the ladder through pipetting to ensure homogeneity. A volume of 6 μl of the marker was directly loaded onto the agarose gel alongside the PCR product samples for molecular weight estimation.

**Agarose Gel Electrophoresis**

Agarose gel electrophoresis was carried out following the method described by **(20)** with minor modifications. Briefly, 1 g of electrophoresis-grade agarose was dissolved in 100 mL of TBE buffer by heating in a microwave oven with intermittent swirling until it was fully dissolved. The molten agarose was cooled to approximately 70°C, after which ethidium bromide was added at a final concentration of 0.5 μg/ml and mixed thoroughly. The warm agarose was then poured into a gel casting tray fitted with an appropriate comb and allowed to solidify at room temperature.

Once the gel had been set, the comb was carefully removed, and the electrophoresis tank was filled with TBE buffer. Twenty microliters of each PCR product, along with the positive and negative controls, were loaded into individual wells of the gel. Electrophoresis was conducted at a constant voltage of 1–5 volts per centimeter of the tank length for approximately 30 minutes. After the run was completed, the gel was transferred to a UV transilluminator for visualization. Gel images were captured using a gel documentation system, and the resulting data were analyzed using computer software.
